# Generalizability Improvement of Interpretable Symbolic Regression Models for Quantitative Structure–Activity Relationships

**DOI:** 10.1021/acsomega.3c09047

**Published:** 2024-02-16

**Authors:** Raku Shirasawa, Katsushi Takaki, Tomoyuki Miyao

**Affiliations:** † Graduate School of Science and Technology, Nara Institute of Science and Technology, 8916-5 Takayama-cho, Ikoma, Nara 630-0192, Japan; ‡ Advanced Research Laboratory, Technology Infrastructure Center, Technology Platform, Sony Group Corporation, Atsugi Tec., 4-14-1 Asahi-cho, Atsugi-shi, Kanagawa 243-0014, Japan; § Data Science Center, Nara Institute of Science and Technology, 8916-5 Takayama-cho, Ikoma, Nara 630-0192, Japan

## Abstract

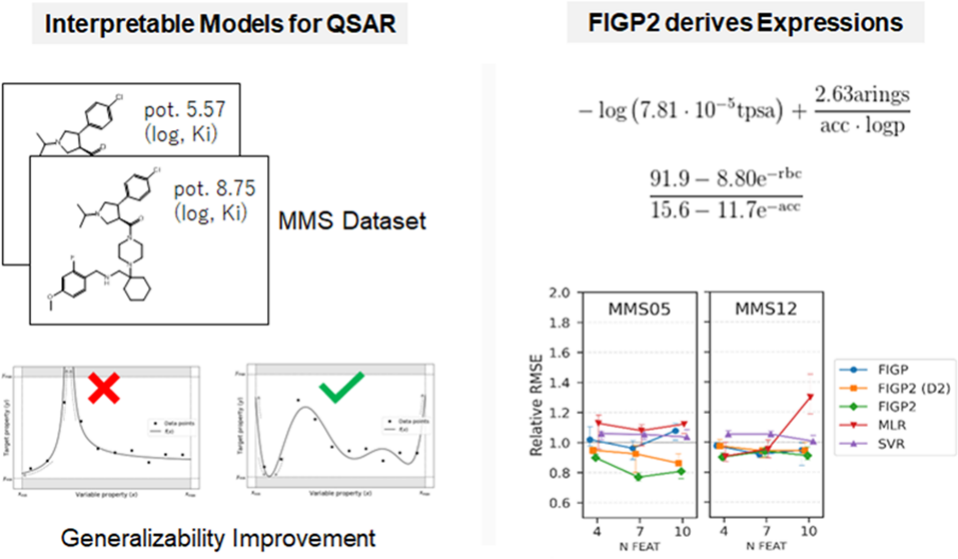

In the pursuit of optimal quantitative structure–activity relationship (QSAR) models, two key factors are paramount: the robustness of predictive ability and the interpretability of the model. Symbolic regression (SR) searches for the mathematical expressions that explain a training data set. Thus, the models provided by SR are globally interpretable. We previously proposed an SR method that can generate interpretable expressions by humans. This study introduces an enhanced symbolic regression method, termed filter-induced genetic programming 2 (FIGP2), as an extension of our previously proposed SR method. FIGP2 is designed to improve the generalizability of SR models and to be applicable to data sets in which cost-intensive descriptors are employed. The FIGP2 method incorporates two major improvements: a modified domain filter to eradicate diverging expressions based on optimal calculation and the introduction of a stability metric to penalize expressions that would lead to overfitting. Our retrospective comparative analysis using 12 structure–activity relationship data sets revealed that FIGP2 surpassed the previously proposed SR method and conventional modeling methods, such as support vector regression and multivariate linear regression in terms of predictive performance. Generated mathematical expressions by FIGP2 were relatively simple and not divergent in the domain of function. Taken together, FIGP2 can be used for making interpretable regression models with predictive ability.

## 1 Introduction

The interpretation of quantitative structure–activity relationship (QSAR) models tries to unveil the characteristics of prediction models in a way that humans can understand.^1,2^ Recently, it has also been termed as an explainable artificial intelligence technique,^3,4^ mostly in combination with deep neural networks. Interpretation approaches can be classified into two categories: global and local, corresponding to focusing on the prediction model itself and input instances, respectively. Various studies were reported on the local interpretation methods and associated applications, such as the identification of important substructures (atoms) in a molecule,^5−9^ compound optimization^10,11^ and finding a potential artifact of a model.^12^ On the other hand, methods for global interpretations are limited. One obvious method is to employ interpretable machine learning methods.^13^ Among them, multivariate linear regression (MLR) using meaningful chemical descriptors has been widely used, in particular for interpreting chemical reaction prediction models.^14,15^ Although this simple approach is solid and effective, a fundamental limitation lies in the fact that the model structure is fixed as a linear combination of descriptors.

Symbolic regression (SR) has the potential to overcome this limitation while keeping an interpretable model structure. SR proposes mathematical expressions (formula) fitting to a training data set, and functional forms are simultaneously optimized during training.^16^ Due to the diverse search space of expressions, genetic programming (GP) is usually used as a search algorithm. Not many QSAR or quantitative structure–property relationship (QSPR) studies related to SR are reported, mainly focusing on higher prediction models^17^ or on feature set extraction.^18,19^

For practical QSAR applications, the expressions generated by SR should be simple yet flexible to balance interpretability and predictability. SR expressions fitted to a training data set are sometimes too complicated and/or produce unrealistic values (e.g., infinity) when predicting compounds out of the domain of applicability. To overcome these drawbacks, we previously proposed an SR method known as filter-introduced genetic programming (FIGP), which incorporates three filters: the function filter (F-filter), variable filter (V-filter), and domain filter (D-filter).^20^ V-filter restricts every variable to a single appearance; the F-filter curbs the recursive use of functionals, and the D-filter ensures model output values are in the predefined range of the objective variable. V-filter and F-filter simply restrict the functional search space. However, the D-filter requires many data points (samples) in the descriptor space to cover the domain of the input variables. Apparently, this is impractical when the cost of generating descriptors is high, such as for quantum chemistry-based descriptors. Without sufficient test data, the D-filter may not detect unqualified expressions that potentially yield infinite values. Furthermore, since FIGP uses the fitness to a training data set as the evaluation metric, like many other SR modeling approaches, an intrinsic limitation of overfitting to training data or the lack of generalizability still remains.

In this study, we propose FIGP2, an extension of FIGP that introduces a modified domain filter (D2-filter) and a stability metric (STBL) in the fitness function. D2-filter detects candidate expressions that violate the range of the objective variable. Unlike D-filter, the D2-filter directly maximizes/minimizes the outputs of candidate expressions by optimization techniques. STBL estimates the robustness of an expression against perturbations in both the regression coefficients and independent variables. In FIGP2, this metric is simply added to the fitness of the training data set for proposing expressions with generalizability. As a proof of concept of QSAR modeling, we prepared 12 target-wise data sets of analogous compounds from the ChEMBL database,^21,22^ using matching molecular series (MMS) analysis.^23^ For these 12 data sets, FIGP2 models consistently showed better predictive ability than FIGP, MLR, and support vector regression (SVR) models,^24^ while being simple and nondivergent expressions for the data set domain. Our implementation of FIGP2 is publicly available in the GitHub repository at https://github.com/raku68/FIGP2.

## 2 Materials and Methods

### 2.1 Compound Data Sets

A series of analogous compounds with potency against specific target macromolecules were extracted from the ChEMBL database (version 29)^21,22^ using matching molecular series (MMS) analysis.^23^ From the ChEMBL database, assays against specific human targets with relationship_type: ‘D’ (direct interaction) and confidence_score: 9 (highest confidence) were considered. The potency measurement was the logarithm of inhibition constant (p*K_i_*), and only compounds having potency values with standard_relation: ‘=’ and standard_units: “nM” were retained. When multiple p*K_i_* values were assigned to a single compound, the arithmetic mean was used as the potency value. In the above process, standardized chemical structures of the compounds were employed. The chemical structures of compounds were standardized by removing salts and transforming them to the neutralized form of the compounds using an in-house Python script. In this way, target-wise compound data sets with p*K_i_* were prepared. These data sets were further filtered by removing data sets whose sizes were below 300 after selecting compounds in the 10–90 percentile range in terms of heavy atom count. The remaining 66 target-wise compound data sets were subsequently processed by a matched molecular pair (MMP) analysis based on the isomeric SMILES representations of the chemical structures.

An MMS consists of a series of analogous compounds, where every pair of compounds in the MMS forms an MMP relationship. An MMS can be created by collecting a core structure (scaffold) of MMPs. In this study, a user-contributed module of *mmpa*(25) in RDKit^26^ was used with three restrictions: the upper substituent ratio: 0.35, the upper core size in heavy atom counts: 60, and a single substituent point (terminal substituent). The created MMS were filtered by a minimum potency range of 3.0, and a minimum number of compounds of 40. Compound overlap was manually checked among the MMS, and highly overlapped MMS were removed to avoid potential data biases, resulting in the 12 MMS data sets used in this study, as reported in Table 1.

**Table 1:** Matching Molecular Series (MMS) Data Sets

MMS ID	target	# CPDs	average potency (std.) [p*K_i_*]	potency range [pK_i_]
01	melanocortin receptor 4	50	7.09 (0.823)	5.57–9.30
02	melanocortin receptor 4	61	6.93 (0.784)	5.11–8.40
03	histamine H3 receptor	53	8.45 (0.653)	6.80–10.17
04	serotonin 6 (5-HT6) receptor	56	7.89 (0.791)	6.00–9.50
05	μ opioid receptor	42	6.58 (0.922)	5.12–8.51
06	κ opioid receptor	83	7.17 (0.974)	5.13–9.22
07	coagulation factor X	61	9.20 (0.963)	6.72–10.7
08	G protein-coupled receptor 44	51	8.30 (0.890)	5.48–9.70
09	adenosine A2b receptor	42	7.20 (0.625)	5.32–9.00
10	adenosine A3 receptor	42	7.51 (0.853)	5.87–9.52
11	tyrosine-protein kinase ABL	75	8.95 (0.942)	6.39–10.7
12	tyrosine-protein kinase ABL	40	8.44 (0.929)	6.67–10.02

### 2.2 Descriptors for Substituents

Ten numerical descriptors that are expected to be associated with biological activity were used, arings (aromatic ring count), logP (octanol–water partition coefficients), rings (ring count), rbc (rotatable bond count), a_heavy (heavy atom count), vdw_vol (van der Waals volume), mw (molecular weight), acc/don (the number of hydrogen bond acceptors/donors) and tpsa (topological polar surface area). Brief definitions of the descriptors and statistic values for them based on the data sets are provided in Table S1 of the Supporting Information. For each compound, descriptors were calculated only using the substituent part of the chemical structure of the compound due to the almost same contribution from the core structure. From the above 10 descriptors, three subsets of descriptors were prepared (Table 2) based on the results of clustering analysis (Table S1) for testing regression model performance while removing descriptors showing similar trends based on correlation coefficients (Table S2).

**Table 2:** Three Feature Sets^a^

feature set name	descriptors
FEAT10	arings, acc, don, a_heavy, logp, rbc, rings, tpsa, vdw_vol, mw
FEAT7	arings, acc, don, logp, rbc, tpsa, mw
FEAT4	logp, rbc, tpsa, mw

a Three distinct feature sets: FEAT10, FEAT7, and FEAT4, respectively, were chosen for performance evaluations. FEAT10 consisted of all ten descriptors, while FEAT7 (FEAT4) consisted of seven (four) descriptors, which were selected based on clusters, as shown in Table S1.

### 2.3 Symbolic Regression (SR) with Constant-Optimized and Filter-Introduced Genetic Programming (FIGP)

#### 2.3.1 GP for SR

The goal of SR is to uncover the mathematical expression of experimental data in the form of regression models, explaining the objective variable *y* (property or activity) from a set of independent variables **x** (descriptors). To explore the enormous space of expressions, GP^16^ is usually used, in which an expression is usually represented as a tree structure. In the GP, candidate expressions are generated by natural evolution-mimicking operations, followed by the evaluation of the expressions and selection with a random operation. This process is iterated until convergence toward an optimal solution. In traditional GP for SR, constant terms in expressions are either randomly assigned or selected from a set of predetermined values. To increase predictive ability of GP, nonlinear least-squares optimization was introduced to optimize the values for constant terms, resulting in higher predictive performance than those of other GP derivatives and ML models.^27^ For simplicity, GP with nonlinear least-squares optimization of constant terms is termed the GP in this study.

#### 2.3.2 Filter-Introduced Genetic Programming (FIGP)

FIGP employs three filters: V-, F-, and D-filters to eliminate undesirable, complicated, and incomprehensible mathematical expressions during expression generation in GP.^20^ V-filter excludes the expressions that contain identical variables in different terms. F-filter removes expressions that have nested operations among certain operators, ‘exp’, ‘log’, ‘sqrt’, ‘square’, and ‘cube’. D-filter eliminates potentially harmful expressions that could lead to invalid operations, such as zero division by testing data points from external compound data. The D-filter simply calculates the outputs for many test data points outside the training data’s domain to determine whether the output values exceed the predefined range of *y*. Our previous analysis revealed that the D-filter was a prerequisite for avoiding generating expressions with producing infinite values.^20^

### 2.4 Proposed Method: FIGP2

FIGP2 is an extension of FIGP by incorporating two novel techniques: D2-filter and the STBL metric in the fitness function.

#### 2.4.1 D2-Filter

**Figure 1:**
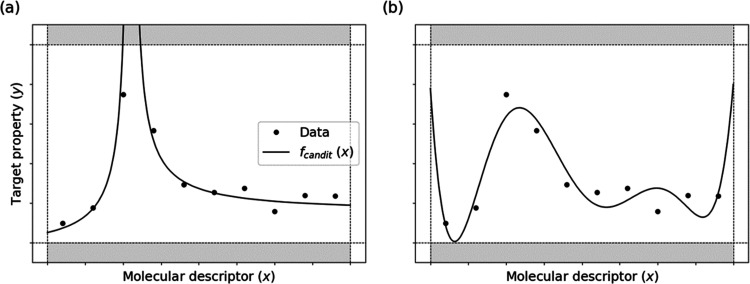
Concept of the optimization-oriented domain filter (D2-filter). The concept of the D2-filter is illustrated. A candidate function whose range violates the predefined target range (*y*) is filtered out (a), while a function whose range is within is kept (b).

The concept of the D2-filter is illustrated in Figure 1. A candidate function whose range violates the predefined range for *y* is filtered out (Figure 1a), while a function whose range is within it is retained (Figure 1b). To detect the maximum/minimum values of expressions in the given domain range, the SciPy optimize library^28^ is used. The maximum and minimum values are determined from five series of optimization calculations. These calculations utilize initial coordinates of **x**, which are randomly selected from the training sets. The target and domain ranges were determined based on the training and test sets in this study.

#### 2.4.2 Stability Metric: STBL

**Figure 2:**
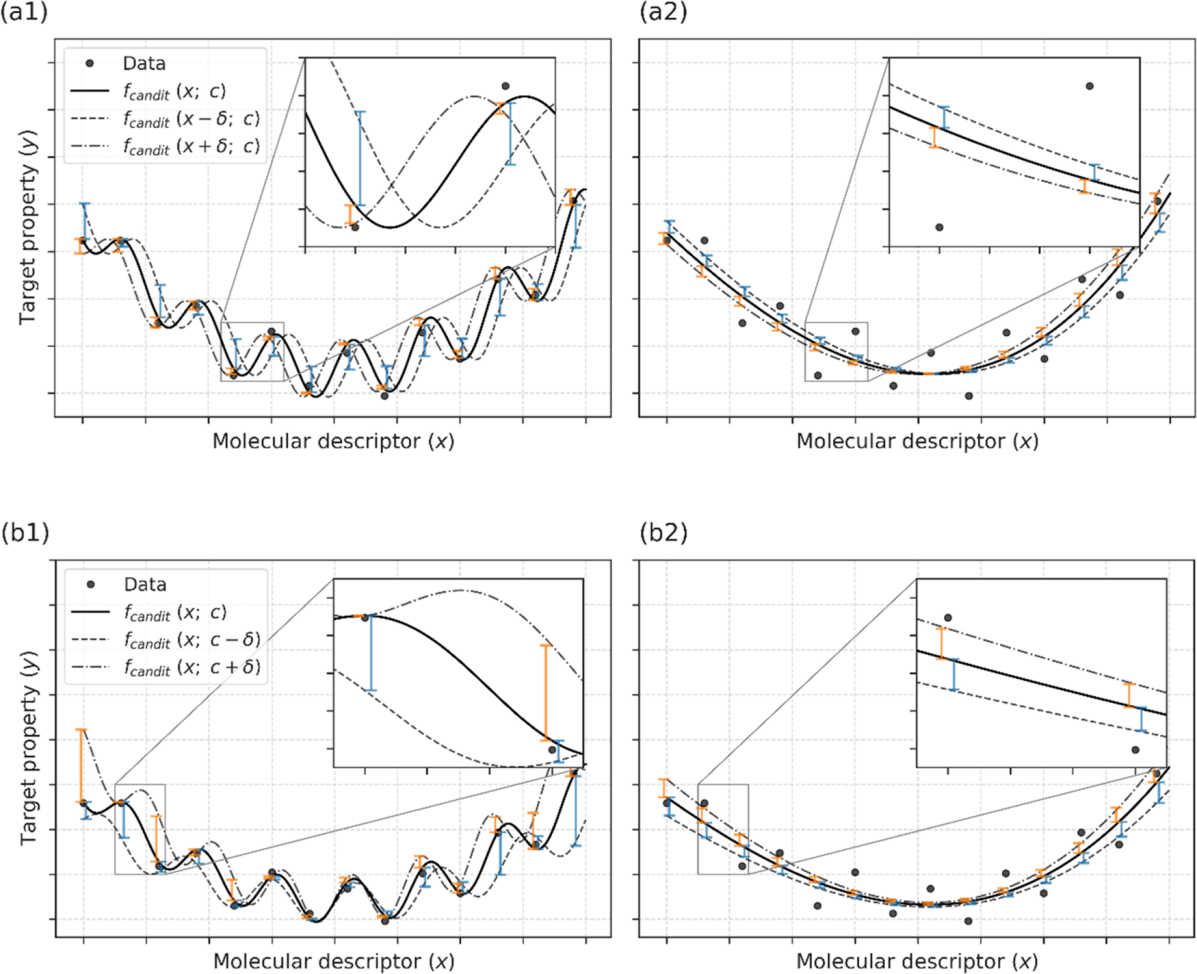
Proposed stability metric: STBL for assessing the robustness against perturbations. Stability metric STBL measures the displacement between fitted- and perturbated functions in the root-mean-squared displacement. Two types of perturbations were considered: descriptor perturbation (a1, a2) and coefficient perturbation (b1, b2). STBL was designed to penalize overfit functions (a1, b1).

As a fitness criterion in GP, the root-mean-square error (RMSE), the coefficient of determination (*R*^2^), or the mean absolute error (MAE) between the observed and predicted *y* values are frequently employed.^16,27^ We propose a stability metric of STBL, which is added to the fitness criterion to improve the generalization performance of the generated expressions. STBL measures displacements between fitted and perturbated functions regarding the root-mean-square displacement (RMSD) (Figure 2). Two types of perturbations are introduced: descriptor perturbations (Figure 2a1,a2) and coefficient perturbations (Figure 2b1,b2). When an expression is represented as f(**x**; **c**), where **c** is a set of coefficients in the expression, STBL for the descriptor perturbation, denoted by STBL_*x*_, is defined as
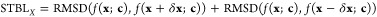
1where δ**x** represents a small perturbation in **x**. Likewise, STBL for the coefficient perturbations, denoted by STBL_c_, is defined as
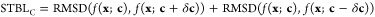
2where δ**c** represents a small perturbation in **c**.

STBL was designed to penalize overfitted expressions (Figure 2a1,2b1) and to encourage the selection of expressions with generalizability. In this study, we used four fitness metrics (FITNESS_0_, FITNESS_X_, FITNESS_C_, and FITNESS_XC_) defined as follows
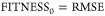
3
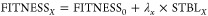
4
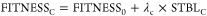
5
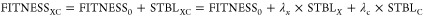
6where λ_*x*_ and λ_c_ are weights for STBL_*x*_ and STBL_c_, respectively.

In this study, we used (λ_*x*_, λ_c_) = (1.0, 1.0) for FITNESS_X_ and FITNESS_C_, and (λ_*x*_, λ_c_) = (0.5, 0.5) for FITNESS_XC_. As the perturbation parameters, we used (δ**x**, δ**c**) = (0.1 × std(*x*), 0.1 × abs(*c*)) for all cases, where std and abs represent the standard deviation and an absolute value, respectively. Prediction performances using other weight combinations are also tested λ_*x*_ (λ_c_): (0.01, 0.02, 0.05, 0.1, 0.2, 0.5), which are mainly reported in Figure S2.

#### 2.4.3 FIGP2 Setting

**Figure 3:**
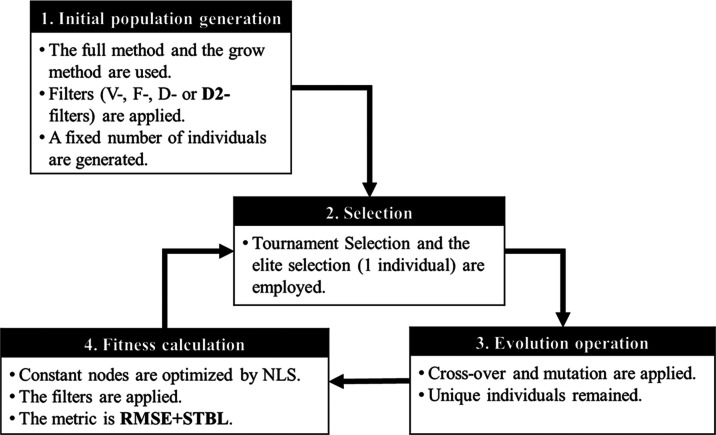
Procedure of FIGP2.

The procedure of FIGP2 comprises four steps, as summarized in Figure 3: the generation of an initial population of individuals (**1**), followed by the selection of individuals for survival (**2**), applying evolutional operations (**3**), and fitness calculation (**4**). Although these steps are almost the same as described in our previous work,^20^ D2-filter in steps 1 and 4, and STBL in step 4, are incorporated in the process. Hyperparameters and set values in both FIGP and FIGP2 are listed in Table 3. In this study, these values were determined based on trial runs using GP. For the conventional GP without any of the FVD filters, the same parameter values as for FIGP2 were used.

**Table 3:** Hyperparameters for FIGP and FIGP2

parameter name	value(s)
population size	200
number of generations	200
tree depth for initial population	1–2
crossover probability	0.7
mutation probability	0.2
tree depth for mutation	0–2
maximum depth	4
function node types	+, −, ×, ÷, sqrt, square, cube, exp, ln
function filter	{sqrt}, {square, cube}, {ln, exp}
tournament size	5
fitness metrics	{RMSE, FITNESS_X_, FITNESS_C_, FITNESS_XC_}
STBL weights (λ_*x*_, λ_c_)	(1.0, 1.0) for FITNESS_X_ and FITNESS_C_, (0.5, 0.5) for FITNESS_XC_
perturbations (δ*x*, δ*c*)	(0.1 × std(*x*), 0.1 × abs(*c*))

### 2.5 Comparison Methods

MLR and SVR^24^ with a nonlinear radial basis function (RBF) kernel were employed as comparison methods. SVR-RBF is commonly used as a nonlinear QSAR model,^29,30^ and MLR is widely used for model interpretation. The loss function of an SVR model is the sum of the norms of the coefficient vectors and the soft margin loss, which leads to robust models even in the presence of outliers. Hyperparameters C, ε, and γ in SVR-RBF were optimized by 5-fold cross-validation using the training data set.

### 2.6 Evaluation Metrics

The prediction performance was evaluated in terms of the RMSE for test data sets, which are isolated from the training phase. Each MMS data set was subjected to an experimental setup consisting of three components: a feature set derived from {FEAT4, 7, and 10}, a training ratio chosen from {0.2, 0.5, and 0.8}, and a pair of training and test data sets from five randomly split data sets. To compare the performance of the methods across each MMS data set, the median value from a total of 45 executions was used. This approach was deemed more suitable than using average values, which in some instances were not representative due to significant score deviations in different experimental settings (average scores are provided in Table S3 for reference).

### 2.7 Software and Implementation of FIGP2

FIGP was implemented on top of the DEAP GP library.^31^ The code for FIGP2 with V-, F-, D-, D2-filters and an STBL-based fitness function is publicly available in a GitHub repository at https://github.com/raku68/FIGP2 along with example Jupyter notebooks.

## 3 Results and Discussion

FIGP2 was compared with FIGP and standard ML modeling methods: MLR and SVR. In the following sections, FIGP2 uses D2-filters and the STBL metric on top of F- and V-Filters, FIGP2 (D2) uses a D2-filter and no STBL metric combined, and FIGP uses F-, D- and V-Filter unless otherwise specified. In the following sections, FIGP2 uses both STBLx and STBLc unless otherwise noted.

### 3.1 Predictive Performance of FIGP2

The comparison of predictive performance with various modeling algorithms, as reported in Table 4, revealed that FIGP2 demonstrated superior performance for the ten data sets (MMS01–05, 07–10, and 12), while securing the second-best performance for the remaining two data sets (MMS06 and 11). Only utilizing the D2-filter (FIGP2 (D2) in Table 4) exhibited optimal performance for MMS11 and showed some improvement for most data sets when comparing with FIGP, with the exception of MMS06. SVR worked the best only for MMS06 but was found to have the worst or second-worst performance for most of the data sets (MMS01, 02, 04, 05, 07, 10–12). Taken together, the FIGP2 methods have shown significant improvement on previously proposed methods: FIGP and standard ML methods (MLR and SVR). Furthermore, in FIGP, sets of filter and metric combinations were tested (four filters: F-, V-, D-, and D2-filters) and two types of metrics in STBL: perturbation on the independent variables (STBL_X) and coefficients (STBL_C). The comprehensive performance in RMSE is reported in the Supporting Information: Figure S1 as box plots and Tables S4 and S5. In conclusion, the applications of the D2-filter in conjunction with the STBL metric (FIGP2) significantly enhanced the predictive performance. However, the implementation of the D2-filter in isolation (FIGP2 (D2)) resulted in only marginal improvements in performance compared to the use of D-filter (FIGP).

**Table 4:** Overall Predictive Performance in the Median RMSE

MMS ID	FIGP	FIGP2 (D2)^a^	FIGP2	MLR	SVR
01	1.16	1.08	**1.06**	1.39	1.09
02	0.91	0.83	**0.81**	0.86	0.87
03	1.00	0.96	**0.838**	1.03	0.840
04	0.98	0.95	**0.90**	0.92	1.06
05	1.04	0.94	**0.85**	1.12	1.07
06	0.99	1.00	0.99	1.02	**0.95**
07	0.98	0.96	**0.86**	0.94	1.06
08	1.21	1.103	**1.099**	1.19	1.102
09	1.20	1.09	**1.05**	1.21	1.14
10	0.78	0.74	**0.72**	0.85	0.96
11	0.97	**0.92**	0.94	0.97	1.04
12	1.04	1.05	**1.01**	1.10	1.15

a FIGP2 (D2): FIGP2 with F-, V-, and D2-Filters.

### 3.2 Sensitivity of Model Performance to the Number of Descriptors and Training Data Set Size

**Figure 4:**
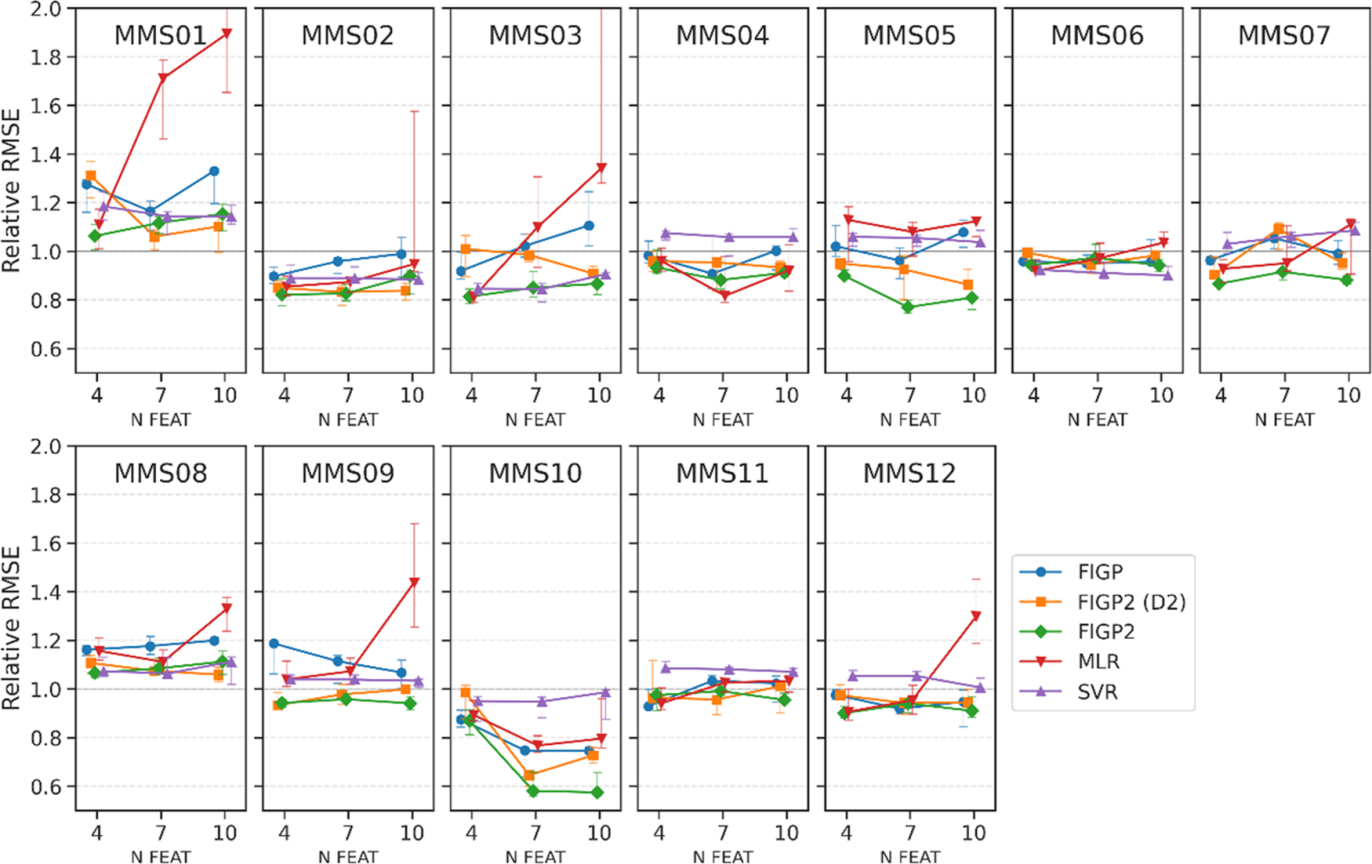
Sensitivity of model performance to the number of descriptors in RMSE. Relative RMSE values for the test data sets of the five methods: FIGP, FIGP2 (D2), and FIGP2, MLR, and SVR are plotted against the number of features (N FEAT). These values were normalized by the RMSE values by the mean predictor. Each point in a line represents the median value, with error bars indicating the 40th and 60th percentiles.

For five regression methods: FIGP, two FIGP2 methods, MLR, and SVR, predictive performances against the number of descriptors (features) and training data set size were investigated. In terms of the sensitivity of model performance to the number of descriptors, relative RMSE values using the five regression methods in combination with the three feature sets are reported in Figure 4. As a control, the mean predictor, which always outputs the mean value of the training data set, was constructed. The relative RMSE value was calculated as the ratio of the RMSE value using the method to that using the mean predictor. The three feature sets in Table 2 were represented as the number of features (N FEAT in Figure 4). In Figure 4, for each method, the relative median RMSE values were plotted as a line with error bars representing the 40th and 60th percentiles. For MMS01 and MMS08, all methods performed worse than the mean predictor, indicating that none of the ML methods could produce reliable predictive models. For the rest of the MMS, the FIGP methods overall outperformed SVR (purple in Figure 4), except for MMS06. FIGP (blue) and FIGP2 (D2) (orange) exhibited similar performance for most data sets, with FIGP2 (D2) slightly better for MMS02, 05, and 09. FIGP2 (green) achieved the best scores for MMS05, 07, 09, and 10 and was comparable to the best for the other MMS. For most methods, the relative RMSE values did not change drastically as N FEAT increased, except for MMS05 and 10, where FIGP methods improved predictive ability.

**Figure 5:**
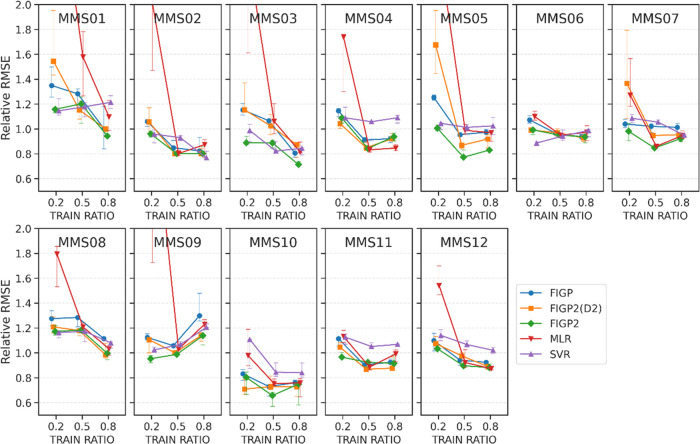
Sensitivity of model performance to the training data set size in RMSE. Relative RMSE values for the test data sets of the five methods: FIGP, FIGP2 (D2), and FIGP2, MLR and SVR are plotted against the training data set size, represented by (TRAIN RATIO). These values were normalized by the RMSE values by the mean predictor. Each point in a line represents the median RMSE value, with error bars indicating the 40th and 60th percentiles. Three training data set sizes: 0.2, 0.5, and 0.8 were tested.

As regards the sensitivity to the training data set size, Figure 5 reports the prediction performance of the five methods against different training data sizes, which were represented as the ratios: 0.2, 0.5, and 0.8 (TRAIN RATIO). The RMSE values were normalized by that by using the mean predictor, like we did for the sensitivity analysis against the number of descriptors. Overall, for the smallest TRAIN RATIO (0.2), MLR performed poorly (red in Figure 5), and FIGP2 (D2) was also worse than the other 3 methods. When making SR models using a limited number of data points in combination with a D2-filter, it might be important to introduce STBL. The performances of MLR and FIGP2 improved as the TRAIN RATIO increased (0.5 or 0.8) in most data sets, except for MMS09. FIGP2 consistently performed well compared to the rest of the tested methods. The smallest TRAIN RATIO (0.2), where training data sizes were between 8 and 16, may be insufficient for making effective models. On the other hand, at the largest TRAIN RATIO (0.8), FIGP2 methods produce the best predictive models in most cases, except for MMS02, 04, and 11. Taken together, FIGP2 was better than other ML methods in these two control calculations. The performance of FIGP2 was not sensitive to the number of descriptors chosen in this study (Table 2). Furthermore, STBL in the fitness function of FIGP2 was necessary when the data set size was extremely small.

### 3.3 Mathematical Expressions Derived by SR Methods

#### 3.3.1 Best-Performing Expression in Combination with Various Filters for Each MMS Data Set

Filters played a central role in FIGP2, and the STBL metric improved the generalizability of the produced expressions, as shown in the previous sections. Herein, filters in SR were investigated: combinations of filters and metrics (four filters: F-, V-, D-, and D2-filters and two metrics in STBL: perturbation on the independent variables (STBL_X) and coefficients (STBL_C)). Table 5 presents the best-performing expression derived by the SR method with the filters for each data set. FIGP2 methods (including STBL or D2-filter) yielded the best-performing expressions obtained for 11 data sets, except for MMS01. Among these, ten used the stability metrics (STBL), and four utilized the domain filter (D2-filter). The expressions for MMS07 and 12 were consistent, while the expression for MMS10 did not diverge, as its denominator was invariably greater than zero for e^(−acc)^ ≤ 1.0 (acc ≥ 0). The expression for MMS04 was also consistent, as the range of each variable calculated from the data set was greater than zero, although the interior of the square root could potentially be negative (e.g., arings·don = 0 and logp < 0). Conversely, seven expressions derived without the domain filter could diverge when any of their denominators approached zero (MMS01–03, 05, 08, 09, and 11), with only one expression for MMS07 being continuous. Consequently, FIGP(2) methods without a domain filter frequently produced diverging functions even if they showed better test set prediction performances.

**Table 5:** Best-Performing Expressions for Each MMS Data Set^a^

MMS ID	best expression	RMSE	filters + metric
01		0.634	FIGP
			NO FILTER
02		0.471	FIGP2
			FV + STBL_C
03		0.472	FIGP2
			FV + STBL_C
04		0.606	FIGP2
			FVD2 + STBL_XC
05		0.658	FIGP2
			FV + STBL_C
06		0.828	FIGP2
			FV + STBL_X
07		0.875	FIGP2
			FVD2 + STBL_XC
08		0.937	FIGP2
			FV + STBL_X
09		0.583	FIGP2
			FV + STBL_X
10		0.564	FIGP2
			FVD2 + STBL_XC
11		0.784	FIGP2
			FV + STBL_C
12		0.774	FIGP2
			FVD2

a The best-performing expressions derived by the SR methods for each data set are displayed. For SR methods, introduced filters are specified in the column filters.

#### 3.3.2 Best-Performing Expressions Derived by FIGP2

**Figure 6:**
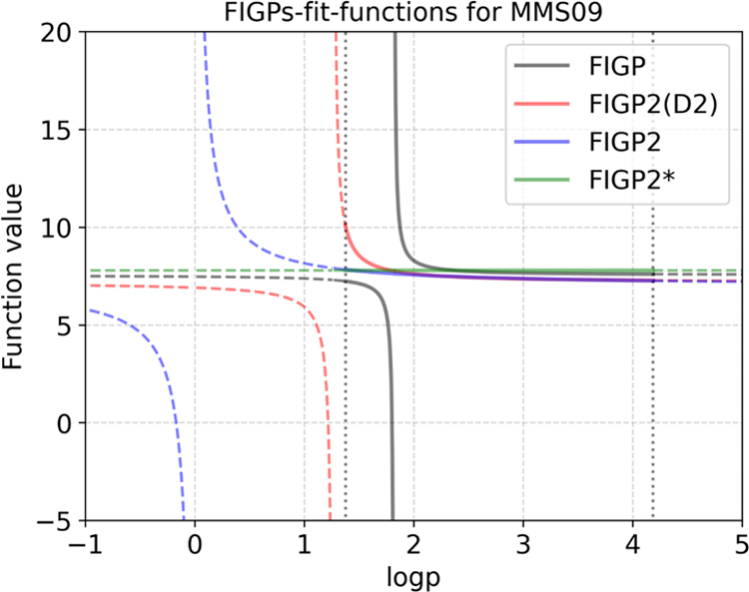
Fit-function derived from FIGPs for MMS09. The best-performing expressions, derived from FIGP, FIGP2(D2), and FIGP2 for MMS09, are depicted along the logp axis. The rest of the variables, e.g., acc, a_heavy, etc., are set to average values. The three expressions are from Table 6, while FIGP2* is expression (7) by generating with an extended logp domain [−1, 5]. Solid lines denote the function values within the logp domain [1.4, 4.2]. Dashed lines indicate values outside this domain.

Focusing on the FIGPs, derived expressions were further scrutinized. Table 6 reports the best-performing expressions for the 12 tested MMS data sets by the three methods: FIGP, FIGP2, and FIGP2 (D2). In terms of predictive performance, FIGP2, FIGP2 (D2), and FIGP produced the best expressions for 67, 25, and 8% of all of the data sets, respectively. The descriptors used in this study (Table 2) can be categorized into three groups based on their value range: positive (a_heavy, vdw_vol, mw > 0), non-negative (arings, acc, don, rbc, rings, tpsa ≥ 0), and any (logp could be negative, zero, or positive). Previously proposed D-filter in FIGP only assured the output values being within the range for tested data points. For example, if a compound having a logarithmic coefficient of 0 is not found in the tested data points, the zero division by the descriptor value might occur for unseen test compounds. The domain of **x** was determined based on the data set ranges, thus some FIGP2 expressions in Table 6 contains such invalid terms as shown in the expression for MMS09: . This limitation can be easily overcome by setting a reasonable domain of descriptors. For example, when setting a range for logp to [−1, 5] for MMS09, the optimized expression by FIGP2 became
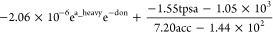
7(RMSE: 0.621/ΔRMSE: −0.054), setting a logically accessible domain for FIGP2 was reasonable to ensure the validity of the expressions. In this study, the domain of the applicability of SR expressions was set to the range of the training data set; thus, the training data set range was used as the domain. Based on the three descriptor groups, expressions derived by FIGP could diverge or have undefined point(s), such as when one of the denominators approached zero (MMS09:  and MMS12: ). On the other hand, all expressions derived from FIGP2 were guaranteed to be consistent within the domain of each data set. Figure 6 illustrates the best-performing expressions derived from the three methods for MMS09, plotted across the logarithmic value range. The expression obtained by FIGP diverges within the logp domain [1.4, 4.2] (as determined from the data set), while FIGP2’s expressions do not. This highlights the efficacy of FIGP2’s D2-filter in discarding divergent functions that might otherwise be slipped through the previously proposed D-filter.

**Table 6:** Best-Performing Expressions Derived from FIGP FVD, FVD2 with or without the Stability Metric^a^

FIGP		
MMS ID	expression	RMSE
01	0.547logp + + 6.90 −	0.734
02	−2.01 × 10^−22^acc·e^tpsa^ + 0.448don + 0.199logp + 5.86	0.63
03	−(3.48 × 10^−5^tpsa − 0.0115)(mw + 13.1rbc + 670) −	**0.498**
04	(0.000450logp + 0.00436)(don·vdw_vol + 1428) −	0.636
05	(0.0831don − 0.0633) + 10.2 −	0.816
06	−0.877don + 0.00974tpsa + (0.00835mw − 1.30)(logp −1.25rbc − 1.16) + 6.50	0.936
07	+ 7.24 +	0.941
08	7.85 +	0.964
09	−0.591a_heavy + 0.0227mw + 0.0227tpsa + + 9.69	0.675
10	0.00376a_heavy(tpsa + e^acc^ − 97.7) + 1.17arings^2^ + logp −0.110rbc + 8.59	0.634
11	+ (0.00163mw − 0.146)(tpsa − 13.8) + 7.62	0.827
12	0.752arings·don − (0.238logp − 0.576)( + 6.24) +	0.844

FIGP2 (D2)		
MMS ID	expression	ΔRMSE
01	log(acc·mw − arings + 539don(logp + 0.859) + 180)	0.114
02	0.794 + 0.339(0.213rbc − 1)^2^(rbc − 4.69) + 5.99	–0.025
03		0.023
04	−(0.0111acc − 0.197)() + 1.50	–0.01
05	arings + 0.804don^3^logp^3^ − 0.0271tpsa + 6.93	–0.068
06		**–0.051**
07	0.781rbc − 0.0151tpsa + 7.10 +	–0.035
08		**–0.013**
09	− 1.12 × 10^−6^e^a_heavy^ + 7.21	–0.054
10	0.348don − 0.457(0.317rbc − 1)^2^ + 6.08 +	–0.047
11	0.935arings^2^ + 0.349logp + (don + 0.00254mw − 1.75) + 8.26	–0.009
12	−acc − logp + 0.00827rbc(vdw_vol − 96.0) + 10	**–0.07**

FIGP2		
MMS ID	expression	ΔRMSE
01		**–0.114**
02	log(109logp × rbc + 4536/)	**–0.097**
03		0.036
04		**–0.03**
05	arings −	**–0.13**
06		0.044
07	0.480rbc − 0.0346tpsa + 9.31	**–0.066**
08	log(1682122mw + 47876122tpsa) − 11.2	–0.01
09		**–0.073**
10		**–0.07**
11		**–0.028**
12		0.015

a For each data set, the best-performing expressions were derived by three symbolic regression methods: FIGP FVD, FIGP (D2), and FIGP2. The scores ΔRMSE for the latter two methods represent the differences from the scores of test set RMSE for FIGP. The best score for each MMS is highlighted in bold.

When comparing the expressions with/without the STBL metric, i.e., FIGP2 (D2) and FIGP2, the expressions from both methods contained similar variable sets but in different compositions. Some expressions by FIGP2 (D2) contained terms that were unstable to variable perturbations, such as an exponential of positive variables (e^a_heavy^ in MMS09), and a higher-degree term of variables (don^3^logp^3^ in MMS05), indicating that the variable perturbation of the STBL metric contributed to eliminating those unstable and potentially overfit functions. On the other hand, the effects of introducing coefficient perturbations (STBL_C) were not clearly observed in the selected expressions, which were expected to contribute to the predictive ability in combination with the variable perturbation.

## 4 Conclusions

Herein, filter-introduced genetic programming 2 (FIGP2) was introduced as an advanced symbolic regression method designed to enhance the generalizability of derived quantitative structure–activity relationship models. FIGP2 improved FIGP in two specific points: introducing a modified domain filter (D2-filter) and a stability metric (STBL). D2-filter effectively eliminates diverging expressions generated during evolution/generation operations without additional unlabeled data points. This is an advantage over the previously proposed D-filter in application to cost-intensive descriptors. The STBL metric is integrated into the fitness function to penalize overfit expressions by evaluating the robustness of expressions in response to variable and coefficient perturbations. Performance comparisons using 12 analogous compound data sets revealed that FIGP2 outperformed conventional machine learning modeling methods: FIGP, support vector regression, and multivariate linear regression. The mathematical expressions generated by FIGP2 were consistent in the training data set domain and simple enough to be interpreted by humans.

As for our future research, the stability of the expression should be pursued. GP-based approaches are intrinsically probabilistic. The proposed expressions are usually different and diverse when different seeds are used. The current FIGP2 cannot overcome this limitation. From a methodological point of view, hyperparameter optimization in FIGP2 studies should be conducted. This will involve optimizing the weights of the stability terms (λ_*x*_, λ_c_) and the perturbation magnitudes (δ**x**, δ**c**), which could potentially enhance the balance between performance and interpretability of the expressions derived from a given data set. Specifically, we plan to conduct several additional parameter sampling executions of FIGP2 prior to actual executions.

We hope that FIGP2 will be used as a modeling method to analyze chemistry-related data sets to extract knowledge, which would support experimental scientists.

## Data Availability

The 12 MMS data sets used in this study are provided in the Supporting Information (MMSDataSets.xlsx), including SMILES strings, potency values, and descriptor values. The python codes for both FIGP and FIGP2 with V-, F-, D-, D2-filters and a STBL-based fitness function are publicly available in a GitHub repository at https://github.com/raku68/FIGP2 along with example notebooks.

## Supporting Information Available

Molecular descriptor profiles (Table S1); correlation coefficients between descriptors (Table S2); overall predictive performance in the average RMSE (Table S3); prediction scores for training sets (Table S4); prediction scores for test sets (Table S5); comparisons of prediction scores with the eight methods for each data set (Figure S1); and comparison of prediction scores with various weights for STBL (Figure S2) (PDF)
MMSDataSets (XLSX)
