# Supplementary material for: Generalizability Improvement of Interpretable Symbolic Regression Models for Quantitative Structure–Activity Relationships
Source: ACS Omega. 2024 Feb 16;9(8):9463–74. doi: 10.1021/acsomega.3c09047 (PMC10905595; doi:10.1021/acsomega.3c09047)
Supplement: Supplementary file 1 — ao3c09047_si_001.pdf [file ao3c09047_si_001.pdf]

Supporting Information

Generalizability Improvement of Interpretable Symbolic Regression Models for Quantitative Structure-activity Relationships

Raku Shirasawa[1,2], Katsushi Takaki[1], and Tomoyuki Miyao[1,3]*

[1]Graduate School of Science and Technology, Nara Institute of Science and Technology, 8916-5 Takayama-cho, Ikoma, Nara, 630-0192, Japan

[2]Advanced Research Laboratory, Technology Infrastructure Center, Technology Platform, Sony Group Corporation, Atsugi Tec., 4-14-1 Asahi-cho, Atsugi-shi, Kanagawa, 243-0014, Japan

[3]Data Science Center, Nara Institute of Science and Technology, 8916-5 Takayama-cho, Ikoma, Nara, 630-0192, Japan

*Corresponding Author:

Email: miyao@dsc.naist.jp

Table S1. Molecular descriptor profiles. For each molecule, 10 descriptors were computed using RDKit[1]. The cluster numbers were determined through KMeans clustering (k=4) of the descriptor values for all 656 molecules, implemented using scikit-learn[2].

Descriptor	Definition	Average (std)	Range [min, max]	Cluster No.
arings	Number of aromatic rings	0.7 (0.6)	[0.0, 2.0]	0
logp	Octanol-water partition coefficient	1.6 (1.2)	[-1.4, 5.3]	0
rings	Number of rings	1.0 (0.6)	[0.0, 3.0]	0
rbc	Rotatable bond counts	1.3 (1.4)	[0.0, 8.0]	1
a_heavy	Number of heavy atoms	9.5 (2.6)	[2.0, 17.0]	2
vdw_vol	Van der waals volume	162.1 (43.7)	[55.7, 304.7]	2
mw	Molecular weight	137.3 (36.4)	[45.1, 253.4]	2
acc	Number of hydrogen bond acceptors	1.1 (0.8)	[0.0, 5.0]	3
doc	Number of hydrogen bond doners	0.5 (0.7)	[0.0, 4.0]	3
tpsa	Topological polar surface area	20.8 (15.6)	[0.0, 83.6]	3

Table S2. Correlation coefficients between descriptors.

	arorings	a_acc	a_don	a_heavy	Logp	RBC	rings	TPSA	vdw_vol	MW
arorings	1.000	-0.272	-0.139	0.536	0.563	-0.174	0.660	-0.203	0.556	0.466
a_acc	-0.272	1.000	0.379	0.150	-0.594	0.507	-0.150	0.681	0.074	0.138
a_don	-0.139	0.379	1.000	0.016	-0.354	0.265	-0.049	0.450	-0.032	-0.029
a_heavy	0.536	0.150	0.016	1.000	0.494	0.401	0.645	0.096	0.973	0.932
logp	0.563	-0.594	-0.354	0.494	1.000	-0.043	0.483	-0.630	0.586	0.499
RBC	-0.174	0.507	0.265	0.401	-0.043	1.000	-0.160	0.257	0.411	0.379
rings	0.660	-0.150	-0.049	0.645	0.483	-0.160	1.000	-0.192	0.652	0.548
TPSA	-0.203	0.681	0.450	0.096	-0.630	0.257	-0.192	1.000	-0.041	0.092
vdw_vol	0.556	0.074	-0.032	0.973	0.586	0.411	0.652	-0.041	1.000	0.920
MW	0.466	0.138	-0.029	0.932	0.499	0.379	0.548	0.092	0.920	1.000

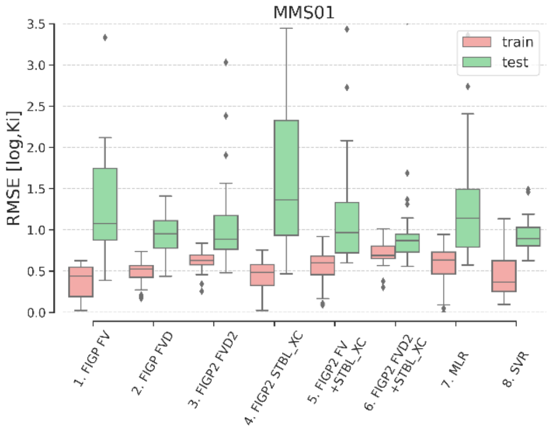
MMS01
RMSE [log,Ki]
train
test
1. FIGP FV
2. FIGP FVD
3. FIGP2 FVD2
4. FIGP2 STBL_XC
5. FIGP2 FV +STBL_XC
6. FIGP2 FVD2 +STBL_XC
7. MLR
8. SVR

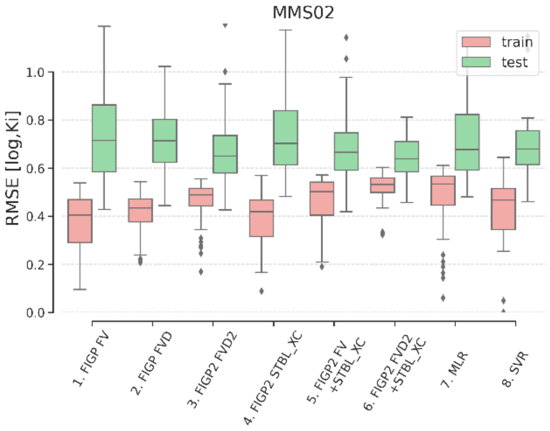
MMS02
RMSE [log,Ki]
train
test
1. FIGP FV
2. FIGP FVD
3. FIGP2 FVD2
4. FIGP2 STBL_XC
5. FIGP2 FV +STBL_XC
6. FIGP2 FVD2 +STBL_XC
7. MLR
8. SVR

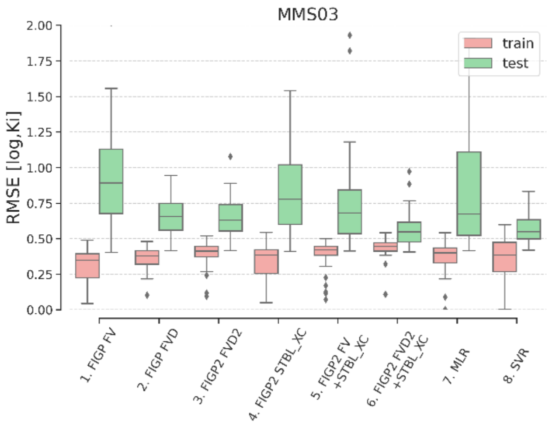
MMS03
RMSE [log,Ki]
train
test
1. FIGP FV
2. FIGP FVD
3. FIGP2 FVD2
4. FIGP2 STBL_XC
5. FIGP2 FV +STBL_XC
6. FIGP2 FVD2 +STBL_XC
7. MLR
8. SVR

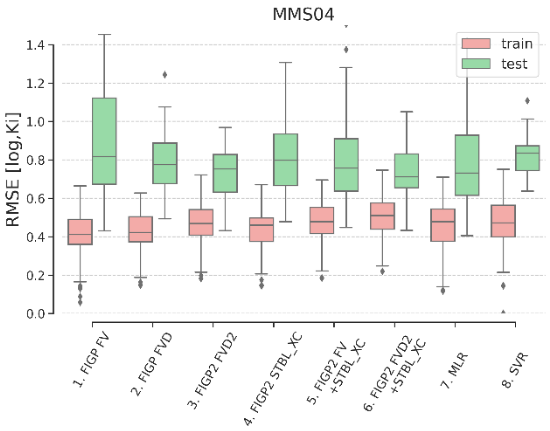
MMS04
RMSE [log,Ki]
train
test
1. FIGP FV
2. FIGP FVD
3. FIGP2 FVD2
4. FIGP2 STBL_XC
5. FIGP2 FV +STBL_XC
6. FIGP2 FVD2 +STBL_XC
7. MLR
8. SVR

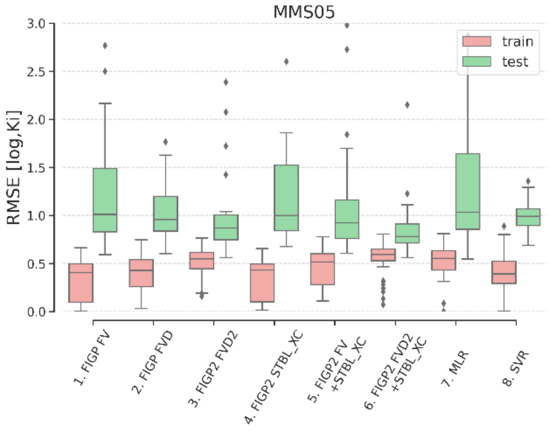
MMS05
RMSE [log,Ki]
train
test
1. FIGP FV
2. FIGP FVD
3. FIGP2 FVD2
4. FIGP2 STBL_XC
5. FIGP2 FV +STBL_XC
6. FIGP2 FVD2 +STBL_XC
7. MLR
8. SVR

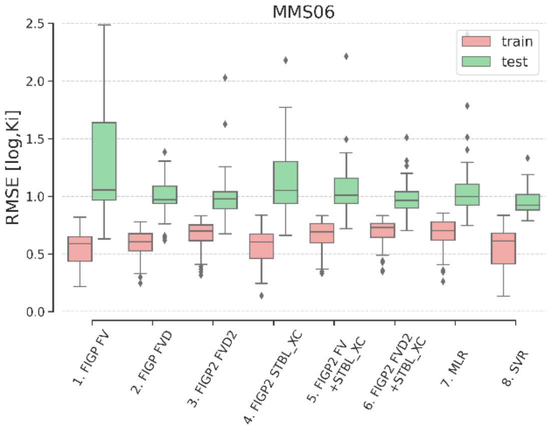
MMS06
RMSE [log,Ki]
train
test
1. FIGP FV
2. FIGP FVD
3. FIGP2 FVD2
4. FIGP2 STBL_XC
5. FIGP2 FV +STBL_XC
6. FIGP2 FVD2 +STBL_XC
7. MLR
8. SVR

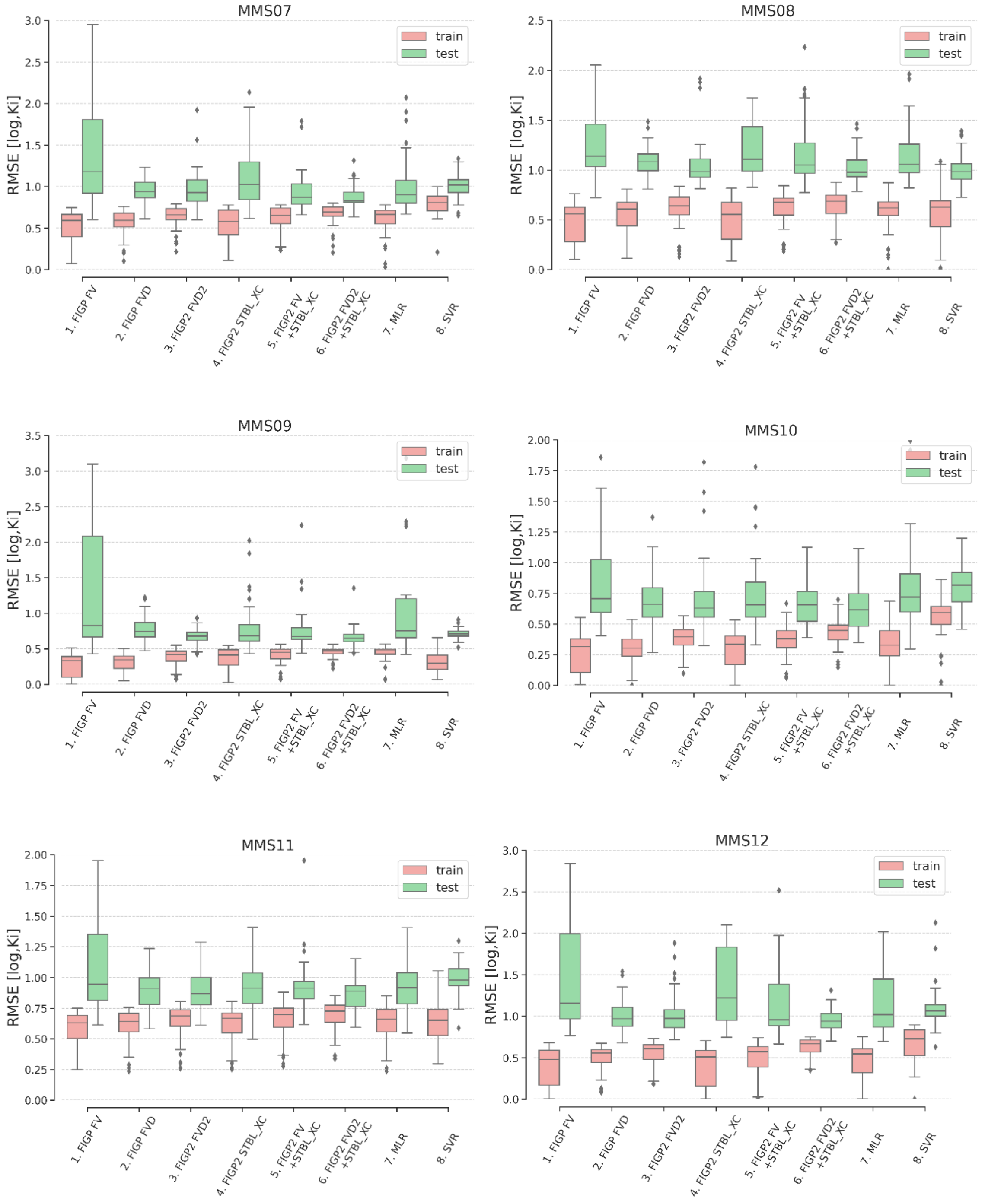

Figure S1. Comparisons of prediction scores with the eight methods for each dataset. Boxplot comparisons of RMSE prediction scores for 12 MMS datasets using 8 methods are displayed. The boxes represent the interquartile range with the median, while the whiskers extend to the furthest datapoint within 1.5 times the interquartile range. Outliers are displayed as individual points. Each method is evaluated for 45 different training/test set splits per dataset. Methods 1–6 are symbolic regressions (FIGPs or

GPSR), while methods 7 and 8 are multiple linear regression (MLR) and support vector regression (SVR). Methods 1 and 2 use FV and FVD filters from Takaki[3], methods 3 and 6 use FVD2 filter, and methods 4–6 use STBL_XC stability metrics for the variables and coefficients. Symbolic regressions and MLR tend to produce overfit models compared to SVR, as evidenced by their superior RMSE scores for the training set but comparable scores for the test set. Notably, symbolic regressions without domain filters (Methods 1, 4 and 5) exhibit larger third quartiles for the test set, indicating that methods with domain filters (Methods 2, 3 and 6) effectively eliminate poorly performing fitted functions. The stability metric STBL_XC also improves model performance by removing overfit functions, as demonstrated by the improved test set scores for Methods 4–6 compared to their counterparts without STBL_XC (Methods 1–3). Overall, the use of both domain filters and the stability metric can yield better results in some cases compared to using only one of these techniques.

Table S3. Overall predictive performance in the average RMSE.

MMS ID	FIGP	FIGP2 (D2)	FIGP2	MLR	SVR
01	1.15	*>10.0*	1.13	*>10.0*	**1.12**
02	0.89	0.86	**0.83**	*2.14*	0.92
03	1.00	*>10.0*	0.94	2.62	**0.88**
04	0.99	1.53	**0.93**	*1.94*	1.06
05	1.11	3.74	**0.92**	*>10.0*	1.07
06	1.02	1.04	1.01	1.09	**0.99**
07	1.00	*>10.0*	**0.91**	1.23	1.03
08	1.22	1.22	1.26	*1.73*	**1.12**
09	1.24	*>10.0*	**1.06**	5.25	1.14
10	0.83	0.99	**0.73**	1.00	0.96
11	0.97	1.01	**0.93**	0.99	*1.05*
12	1.08	1.10	**1.02**	*1.40*	1.1

Table S4. Prediction scores for training sets.

The medians of training set prediction scores for five methods (FIGP, FIGP2 (D2), FIGP2, MLR, and SVR) across 12 datasets are displayed. Each method is evaluated for 45 different training/test splits per dataset. The best value in each row is bolded, while the worst is italicized. FIGP produces the best training set prediction scores for eight datasets (MMS02–04, 06–08, 10 and 11), SVR does so for three datasets (MMS01, 05 and 09), and MLR does so for one dataset (MMS09).

MMS ID	FIGP	FIGP2 (D2)	FIGP2	MLR	SVR
01	0.64	0.77	*0.84*	0.77	**0.45**
02	**0.55**	0.62	0.678	*0.681*	0.6
03	**0.58**	0.63	*0.68*	0.61	0.59
04	**0.53**	0.59	*0.64*	0.60	0.6
05	0.46	0.60	*0.64*	0.60	**0.43**
06	**0.62**	0.72	*0.75*	0.72	0.63
07	**0.62**	0.68	0.72	0.69	*0.84*
08	**0.68**	0.72	*0.77*	0.70	0.7
09	0.56	0.67	0.75	*0.76*	**0.48**
10	**0.36**	0.46	0.52	0.39	*0.7*
11	**0.68**	0.73	*0.77*	0.70	0.69
12	0.60	0.66	0.72	**0.59**	*0.78*

Table S5. Prediction scores for test sets.

The medians of test set prediction scores for five methods (FIGP, FIGP2 (D2), FIGP2, MLR, and SVR) across 12 datasets are displayed. Each method is evaluated for 45 different training/test splits per dataset. The best value in each row is bolded, while the worst is italicized. FIGP2 outperforms other methods in 10 cases (MMS01–05, 07–10, and 12), FIGP2 (D2) does so for MMS11, and SVR does so for MMS06. FIGP performs poorly on most datasets. Overall, FIGP2 effectively eliminates overfit functions and improves predictive models compared to FIGP and FIGP2 (D2), yielding much better or comparable performance to SVR.

MMS	FIGP	FIGP2 (D2)	FIGP2	MLR	SVR
01	1.16	1.08	**1.06**	*1.39*	1.09
02	*0.91*	0.83	**0.81**	0.86	0.87
03	1.00	0.96	**0.838**	*1.03*	0.840
04	0.98	0.95	**0.90**	0.92	*1.06*
05	1.04	0.94	**0.85**	*1.12*	1.07
06	0.99	1.00	0.99	*1.02*	**0.95**
07	0.98	0.96	**0.86**	0.94	*1.06*
08	*1.21*	1.103	**1.099**	1.19	1.102
09	1.20	1.09	**1.05**	*1.21*	1.14
10	0.78	0.74	**0.72**	0.85	*0.96*
11	0.97	**0.92**	0.94	0.97	*1.04*
12	1.04	1.05	**1.01**	1.10	*1.15*

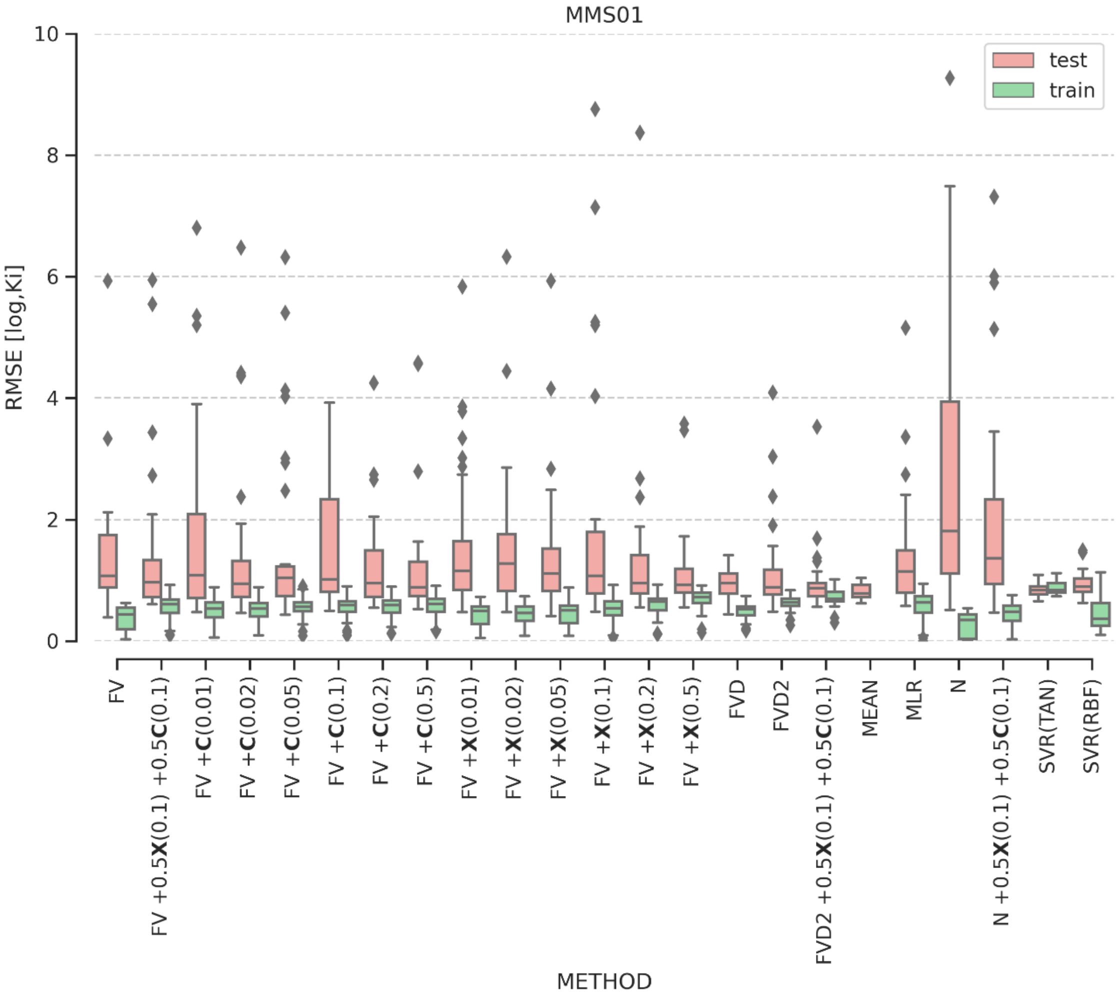
MMS01
RMSE [log,Ki]
10
8
6
4
2
0
test
train
FV
FV +0.5X(0.1) +0.5C(0.1)
FV +C(0.01)
FV +C(0.02)
FV +C(0.05)
FV +C(0.1)
FV +C(0.2)
FV +C(0.5)
FV +X(0.01)
FV +X(0.02)
FV +X(0.05)
FV +X(0.1)
FV +X(0.2)
FV +X(0.5)
FVD
FVD2
FVD2 +0.5X(0.1) +0.5C(0.1)
MEAN
MLR
N
N +0.5X(0.1) +0.5C(0.1)
SVR(TAN)
SVR(RBF)
METHOD

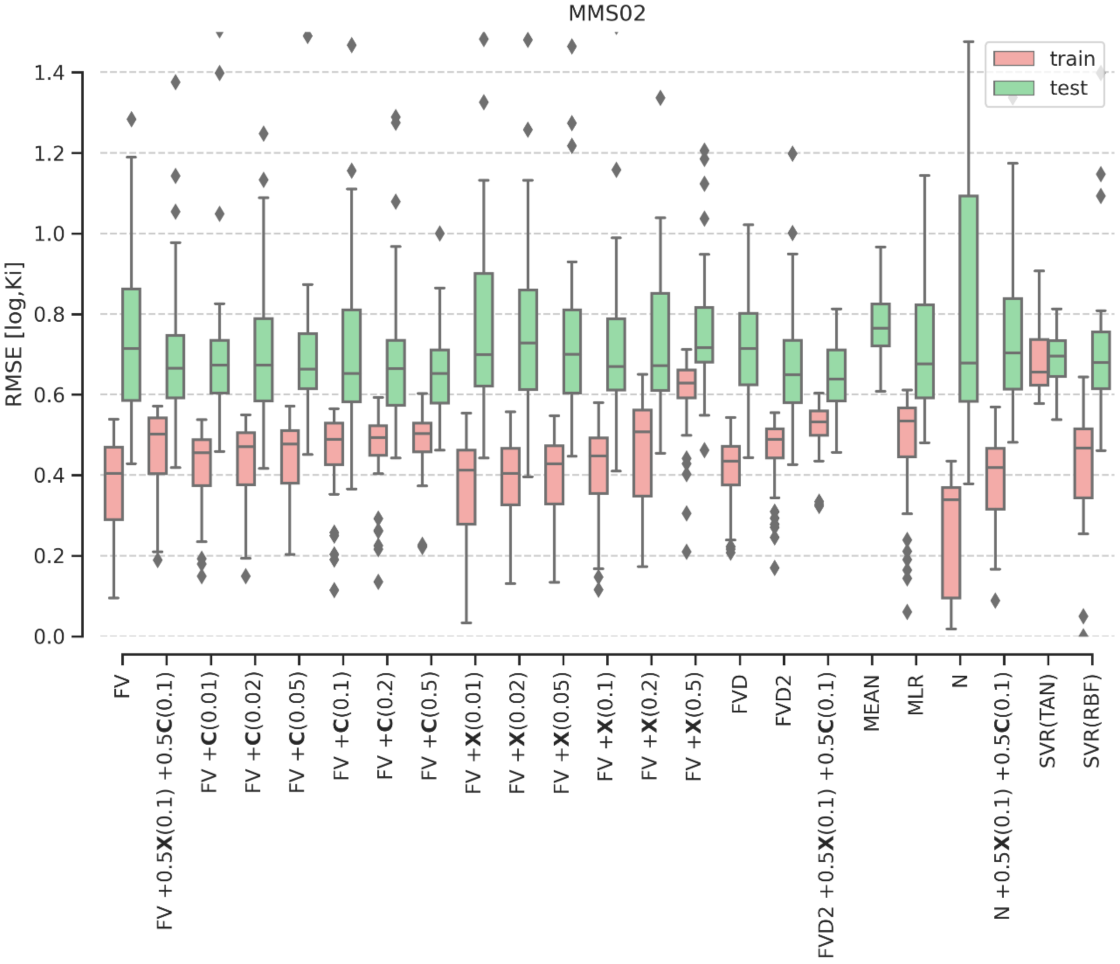
MMS02
train
test
RMSE [log,Ki]
1.4
1.2
1.0
0.8
0.6
0.4
0.2
0.0
FV
FV +0.5X(0.1) +0.5C(0.1)
FV +C(0.01)
FV +C(0.02)
FV +C(0.05)
FV +C(0.1)
FV +C(0.2)
FV +C(0.5)
FV +X(0.01)
FV +X(0.02)
FV +X(0.05)
FV +X(0.1)
FV +X(0.2)
FV +X(0.5)
FVD
FVD2
FVD2 +0.5X(0.1) +0.5C(0.1)
MEAN
MLR
N
N +0.5X(0.1) +0.5C(0.1)
SVR(TAN)
SVR(RBF)
METHOD

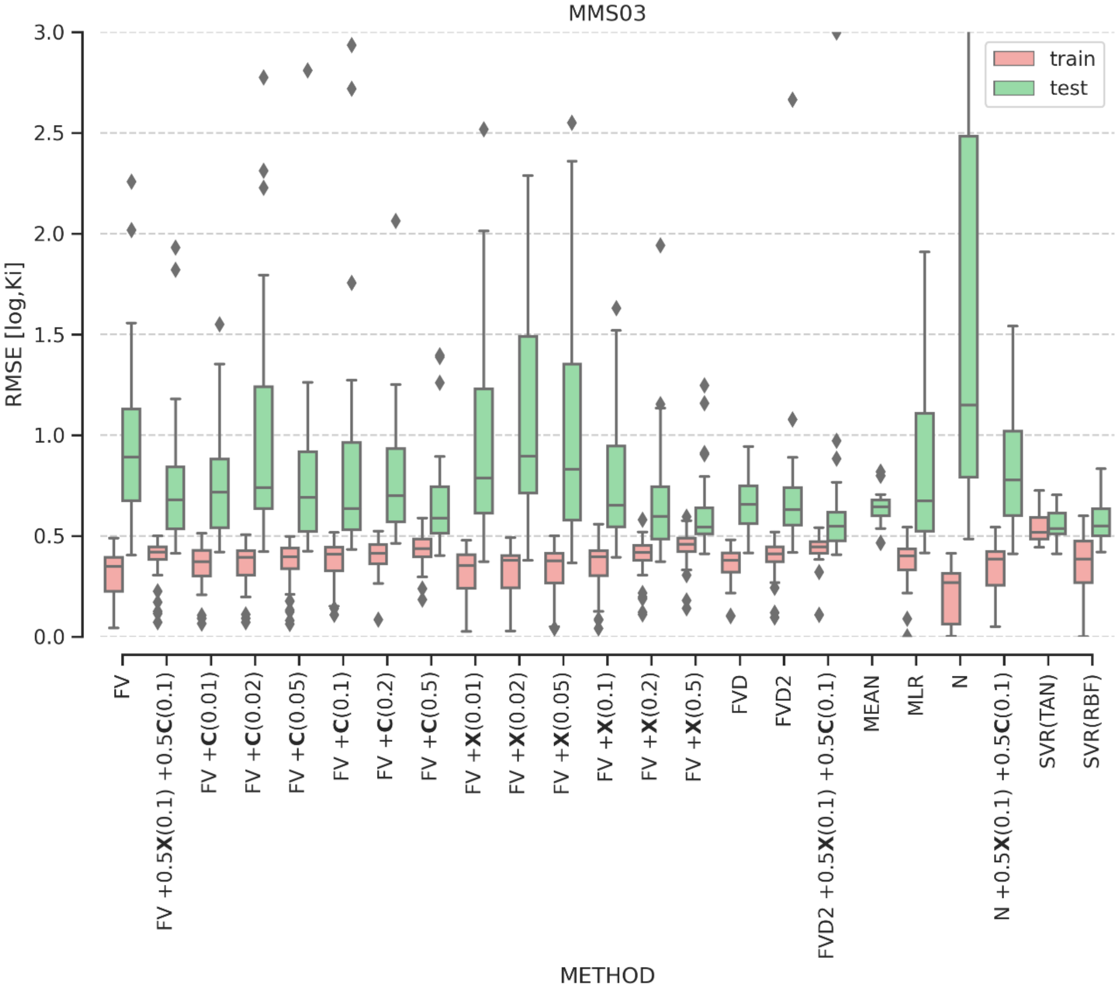
MMS03
train
test
RMSE [log,Ki]
3.0
2.5
2.0
1.5
1.0
0.5
0.0
FV
FV +0.5X(0.1) +0.5C(0.1)
FV +C(0.01)
FV +C(0.02)
FV +C(0.05)
FV +C(0.1)
FV +C(0.2)
FV +C(0.5)
FV +X(0.01)
FV +X(0.02)
FV +X(0.05)
FV +X(0.1)
FV +X(0.2)
FV +X(0.5)
FVD
FVD2
FVD2 +0.5X(0.1) +0.5C(0.1)
MEAN
MLR
N
N +0.5X(0.1) +0.5C(0.1)
SVR(TAN)
SVR(RBF)
METHOD

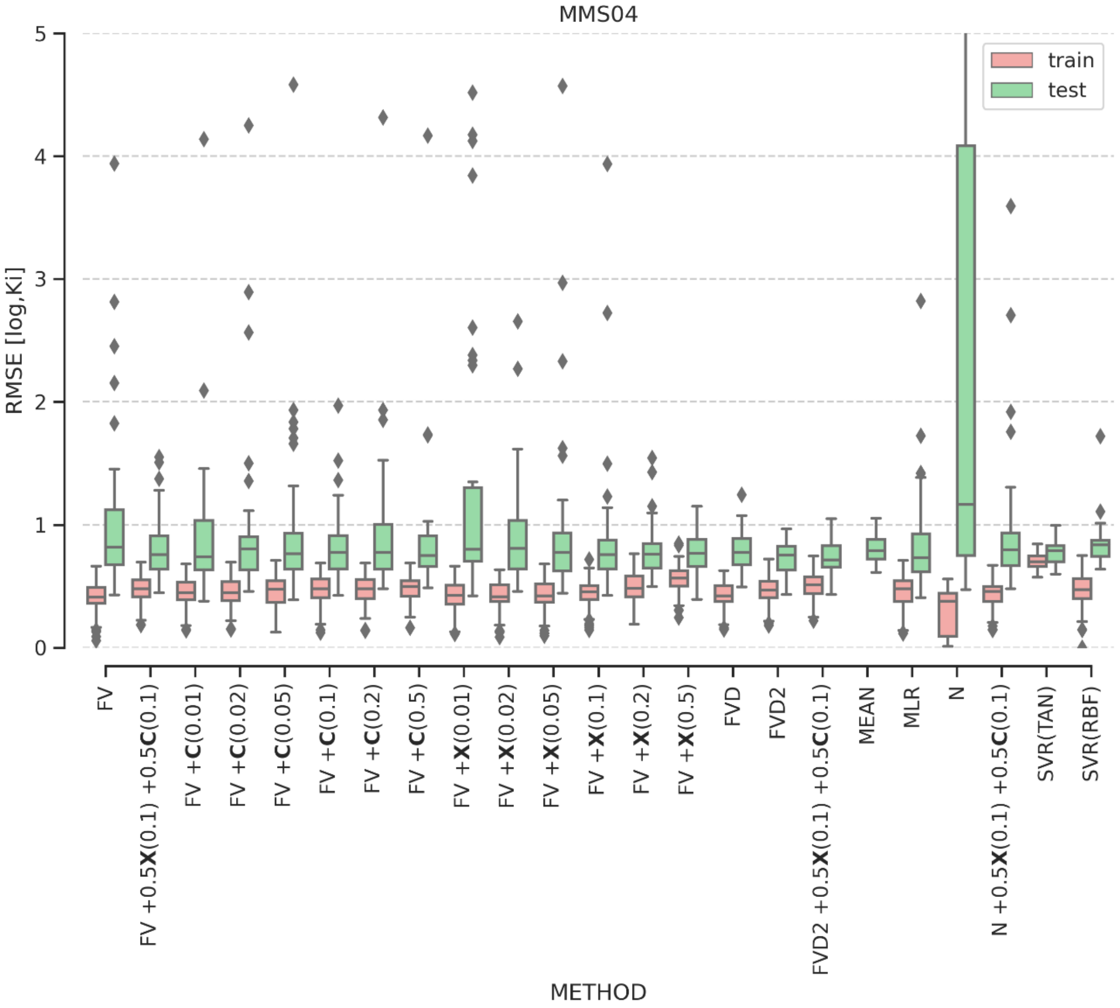
MMS04
RMSE [log,Ki]
METHOD
train
test
FV
FV +0.5X(0.1) +0.5C(0.1)
FV +C(0.01)
FV +C(0.02)
FV +C(0.05)
FV +C(0.1)
FV +C(0.2)
FV +C(0.5)
FV +X(0.01)
FV +X(0.02)
FV +X(0.05)
FV +X(0.1)
FV +X(0.2)
FV +X(0.5)
FVD
FVD2
FVD2 +0.5X(0.1) +0.5C(0.1)
MEAN
MLR
N
N +0.5X(0.1) +0.5C(0.1)
SVR(TAN)
SVR(RBF)

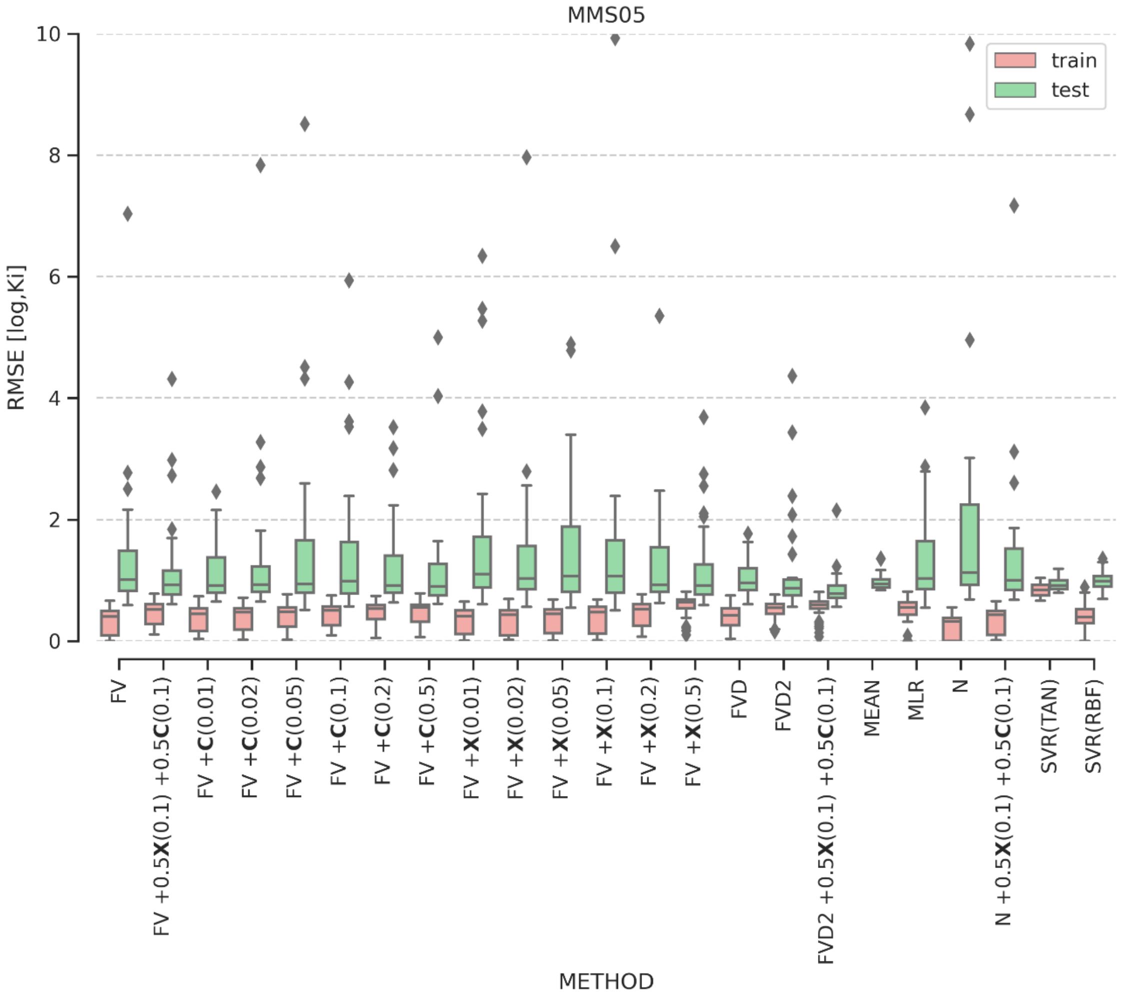
MMS05
train
test
RMSE [log,Ki]
METHOD
FV
FV +0.5X(0.1) +0.5C(0.1)
FV +C(0.01)
FV +C(0.02)
FV +C(0.05)
FV +C(0.1)
FV +C(0.2)
FV +C(0.5)
FV +X(0.01)
FV +X(0.02)
FV +X(0.05)
FV +X(0.1)
FV +X(0.2)
FV +X(0.5)
FVD
FVD2
FVD2 +0.5X(0.1) +0.5C(0.1)
MEAN
MLR
N
N +0.5X(0.1) +0.5C(0.1)
SVR(TAN)
SVR(RBF)

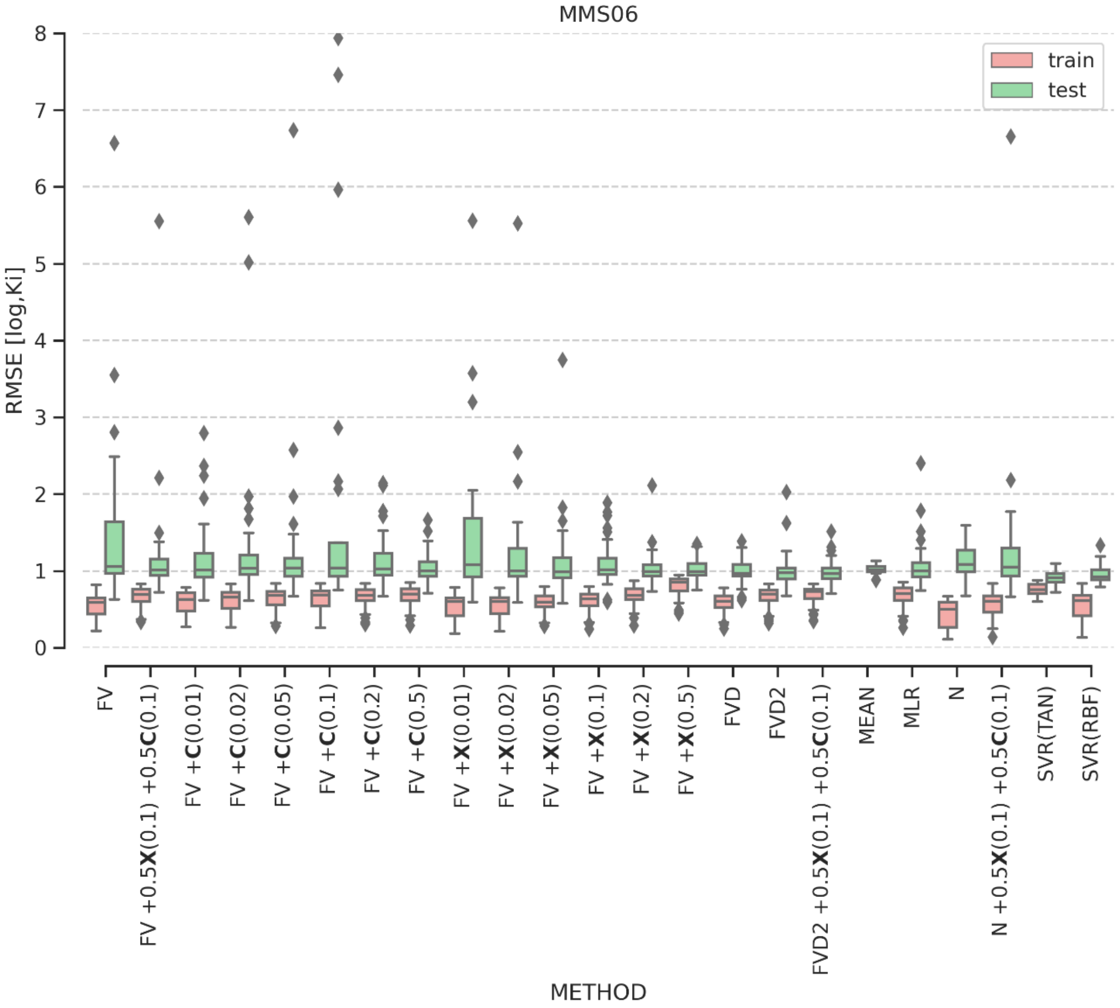
MMS06
RMSE [log,Ki]
train
test
METHOD
FV
FV +0.5X(0.1) +0.5C(0.1)
FV +C(0.01)
FV +C(0.02)
FV +C(0.05)
FV +C(0.1)
FV +C(0.2)
FV +C(0.5)
FV +X(0.01)
FV +X(0.02)
FV +X(0.05)
FV +X(0.1)
FV +X(0.2)
FV +X(0.5)
FVD
FVD2
FVD2 +0.5X(0.1) +0.5C(0.1)
MEAN
MLR
N
N +0.5X(0.1) +0.5C(0.1)
SVR(TAN)
SVR(RBF)

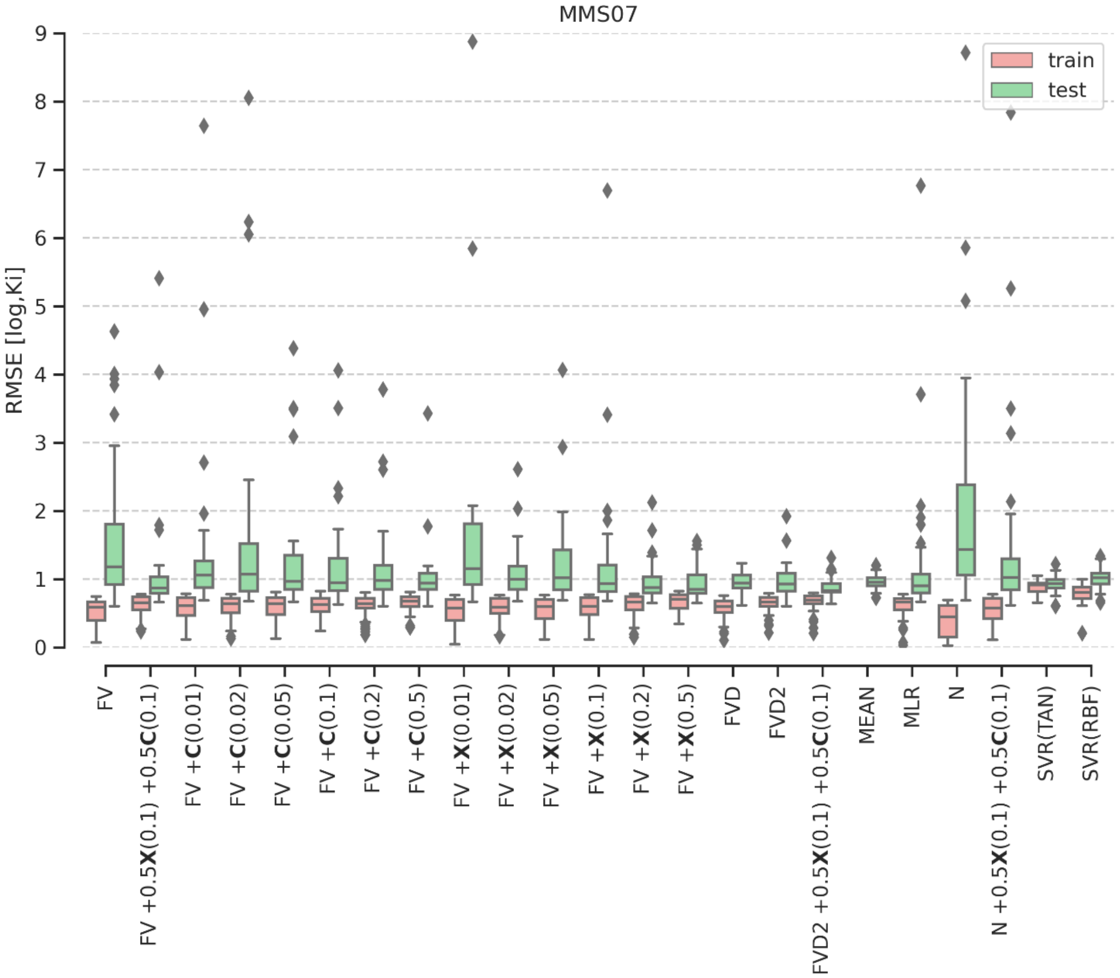
MMS07
RMSE [log,Ki]
train
test
METHOD
FV
FV +0.5X(0.1) +0.5C(0.1)
FV +C(0.01)
FV +C(0.02)
FV +C(0.05)
FV +C(0.1)
FV +C(0.2)
FV +C(0.5)
FV +X(0.01)
FV +X(0.02)
FV +X(0.05)
FV +X(0.1)
FV +X(0.2)
FV +X(0.5)
FVD
FVD2
FVD2 +0.5X(0.1) +0.5C(0.1)
MEAN
MLR
N
N +0.5X(0.1) +0.5C(0.1)
SVR(TAN)
SVR(RBF)

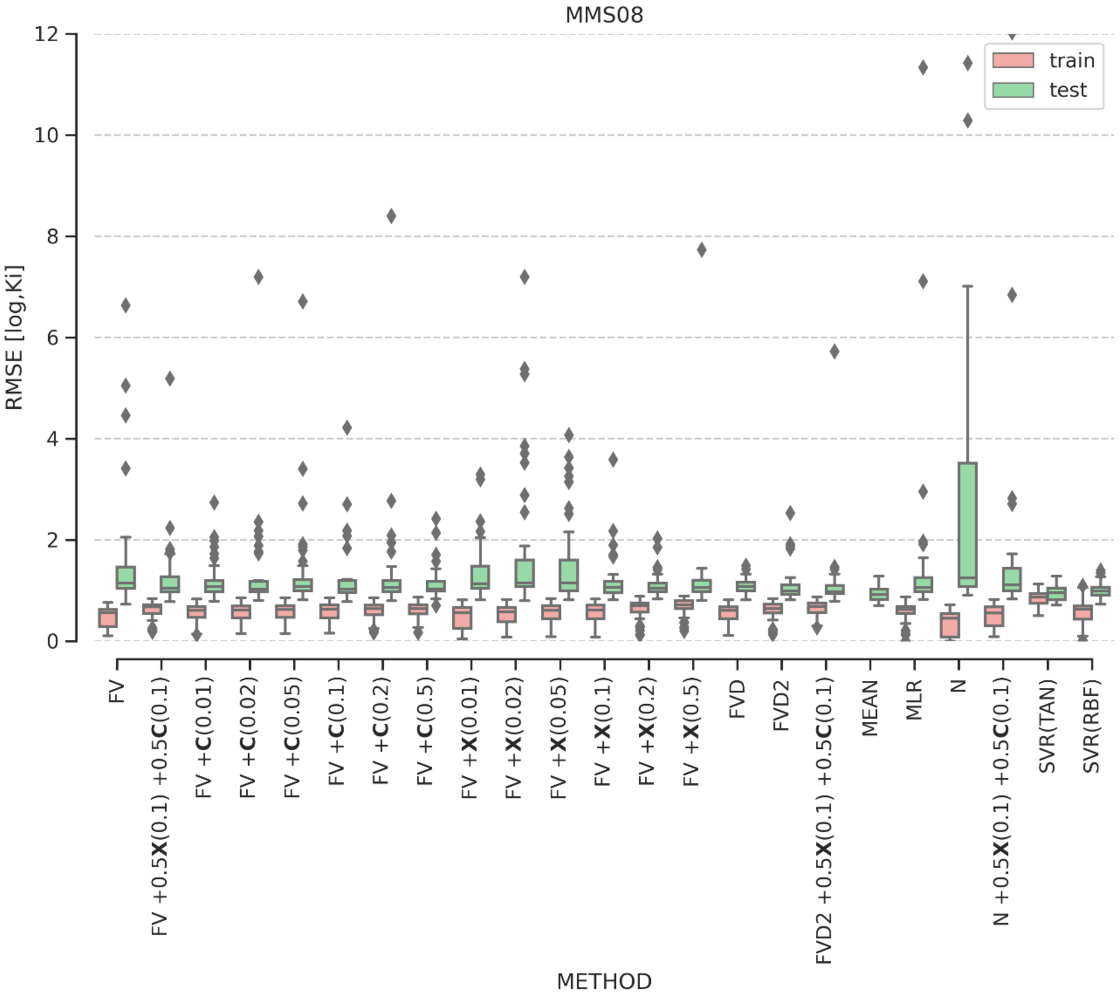
MMS08
RMSE [log,Ki]
0
2
4
6
8
10
12
train
test
FV
FV +0.5X(0.1) +0.5C(0.1)
FV +C(0.01)
FV +C(0.02)
FV +C(0.05)
FV +C(0.1)
FV +C(0.2)
FV +C(0.5)
FV +X(0.01)
FV +X(0.02)
FV +X(0.05)
FV +X(0.1)
FV +X(0.2)
FV +X(0.5)
FVD
FVD2
FVD2 +0.5X(0.1) +0.5C(0.1)
MEAN
MLR
N
N +0.5X(0.1) +0.5C(0.1)
SVR(TAN)
SVR(RBF)
METHOD

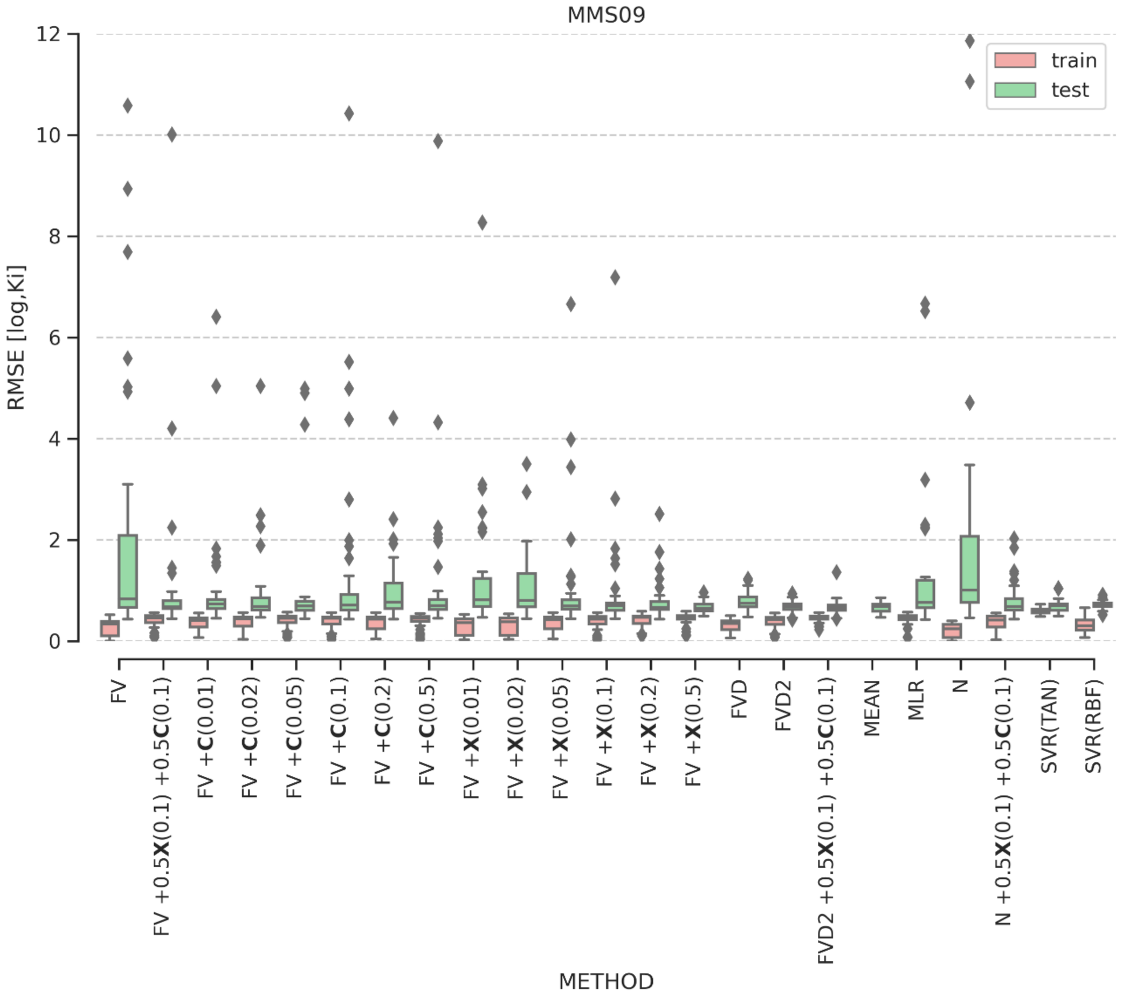
MMS09
RMSE [log,Ki]
train
test
METHOD
FV
FV +0.5X(0.1) +0.5C(0.1)
FV +C(0.01)
FV +C(0.02)
FV +C(0.05)
FV +C(0.1)
FV +C(0.2)
FV +C(0.5)
FV +X(0.01)
FV +X(0.02)
FV +X(0.05)
FV +X(0.1)
FV +X(0.2)
FV +X(0.5)
FVD
FVD2
FVD2 +0.5X(0.1) +0.5C(0.1)
MEAN
MLR
N
N +0.5X(0.1) +0.5C(0.1)
SVR(TAN)
SVR(RBF)

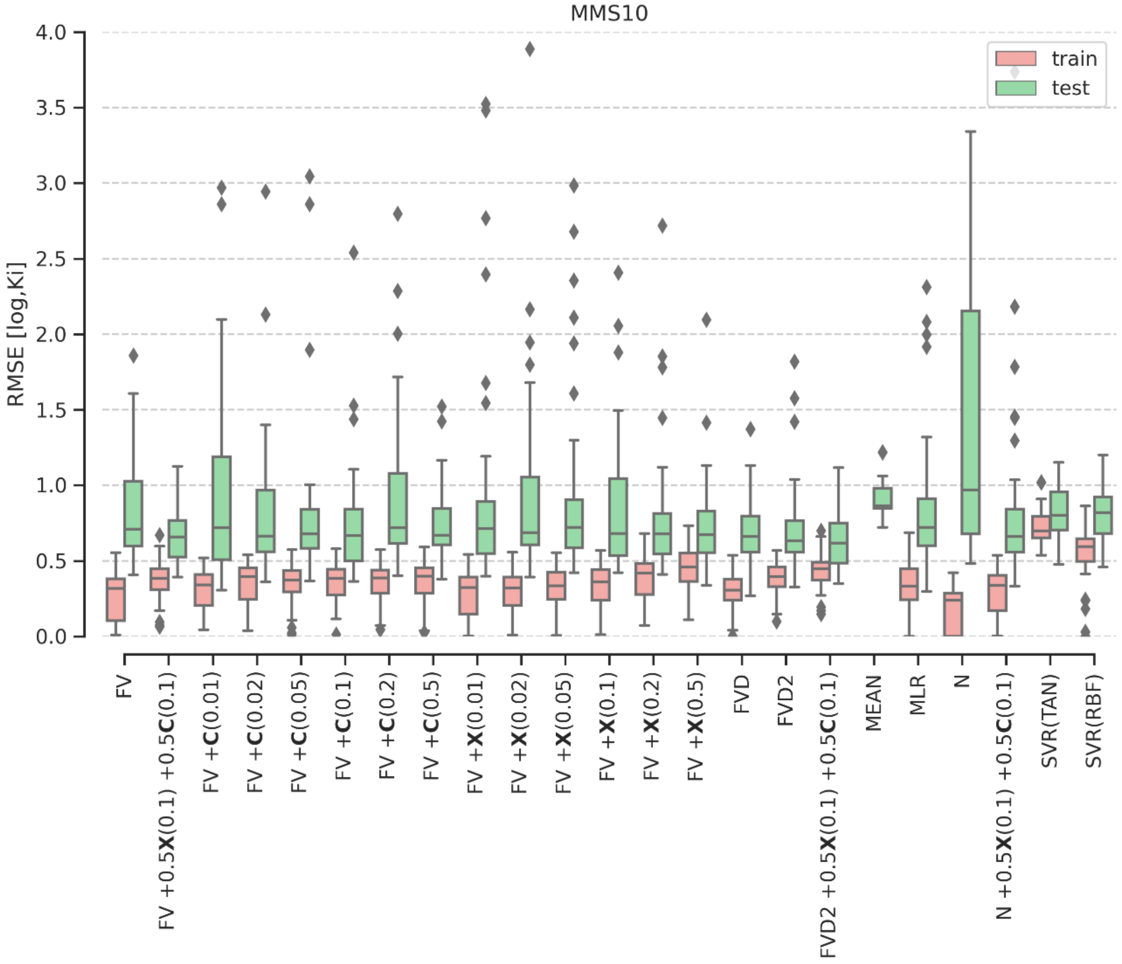
MMS10
RMSE [log,Ki]
4.0
3.5
3.0
2.5
2.0
1.5
1.0
0.5
0.0
train
test
FV
FV +0.5X(0.1) +0.5C(0.1)
FV +C(0.01)
FV +C(0.02)
FV +C(0.05)
FV +C(0.1)
FV +C(0.2)
FV +C(0.5)
FV +X(0.01)
FV +X(0.02)
FV +X(0.05)
FV +X(0.1)
FV +X(0.2)
FV +X(0.5)
FVD
FVD2
FVD2 +0.5X(0.1) +0.5C(0.1)
MEAN
MLR
N
N +0.5X(0.1) +0.5C(0.1)
SVR(TAN)
SVR(RBF)
METHOD

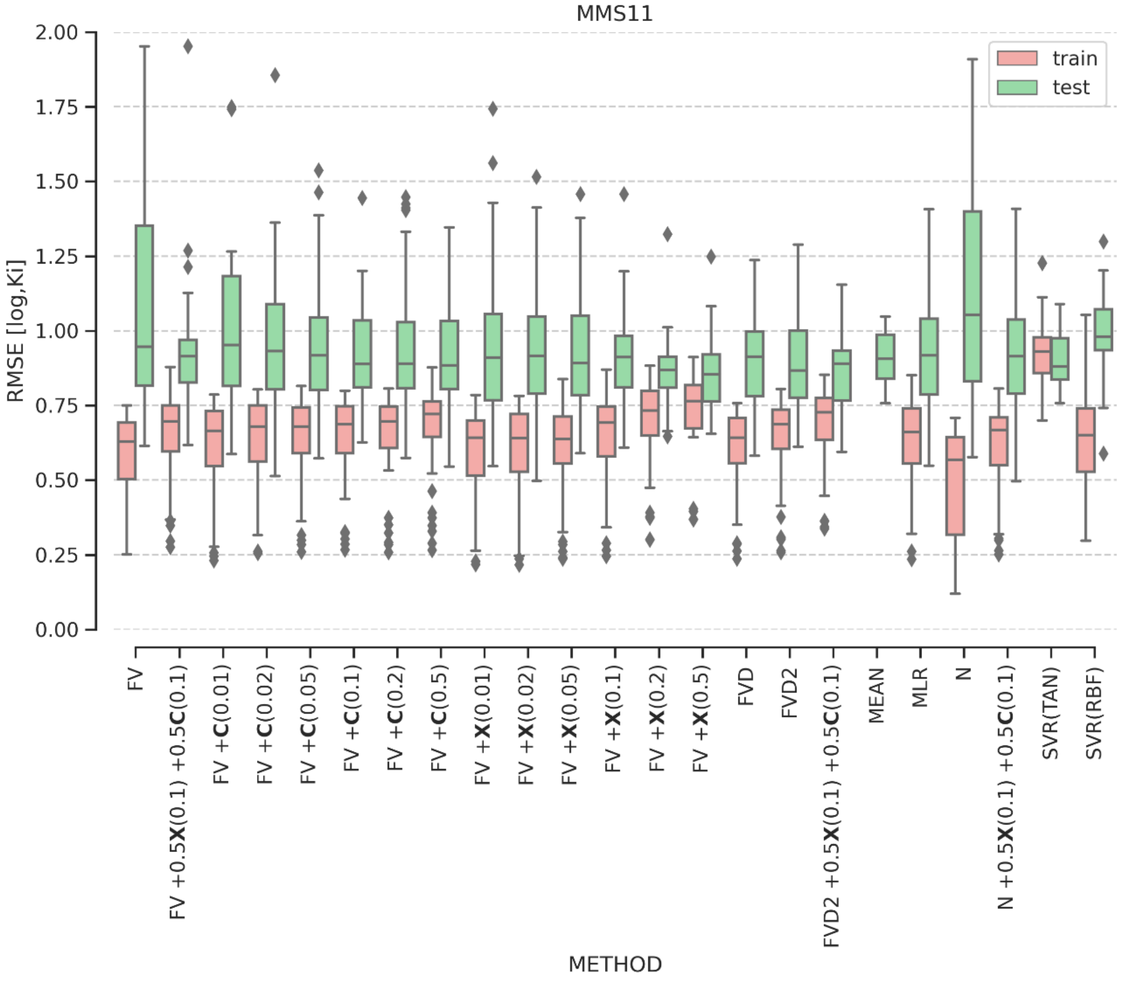
MMS11
train
test
RMSE [log,Ki]
2.00
1.75
1.50
1.25
1.00
0.75
0.50
0.25
0.00
FV
FV +0.5**X**(0.1) +0.5**C**(0.1)
FV +**C**(0.01)
FV +**C**(0.02)
FV +**C**(0.05)
FV +**C**(0.1)
FV +**C**(0.2)
FV +**C**(0.5)
FV +**X**(0.01)
FV +**X**(0.02)
FV +**X**(0.05)
FV +**X**(0.1)
FV +**X**(0.2)
FV +**X**(0.5)
FVD
FVD2
FVD2 +0.5**X**(0.1) +0.5**C**(0.1)
MEAN
MLR
N
N +0.5**X**(0.1) +0.5**C**(0.1)
SVR(TAN)
SVR(RBF)
METHOD

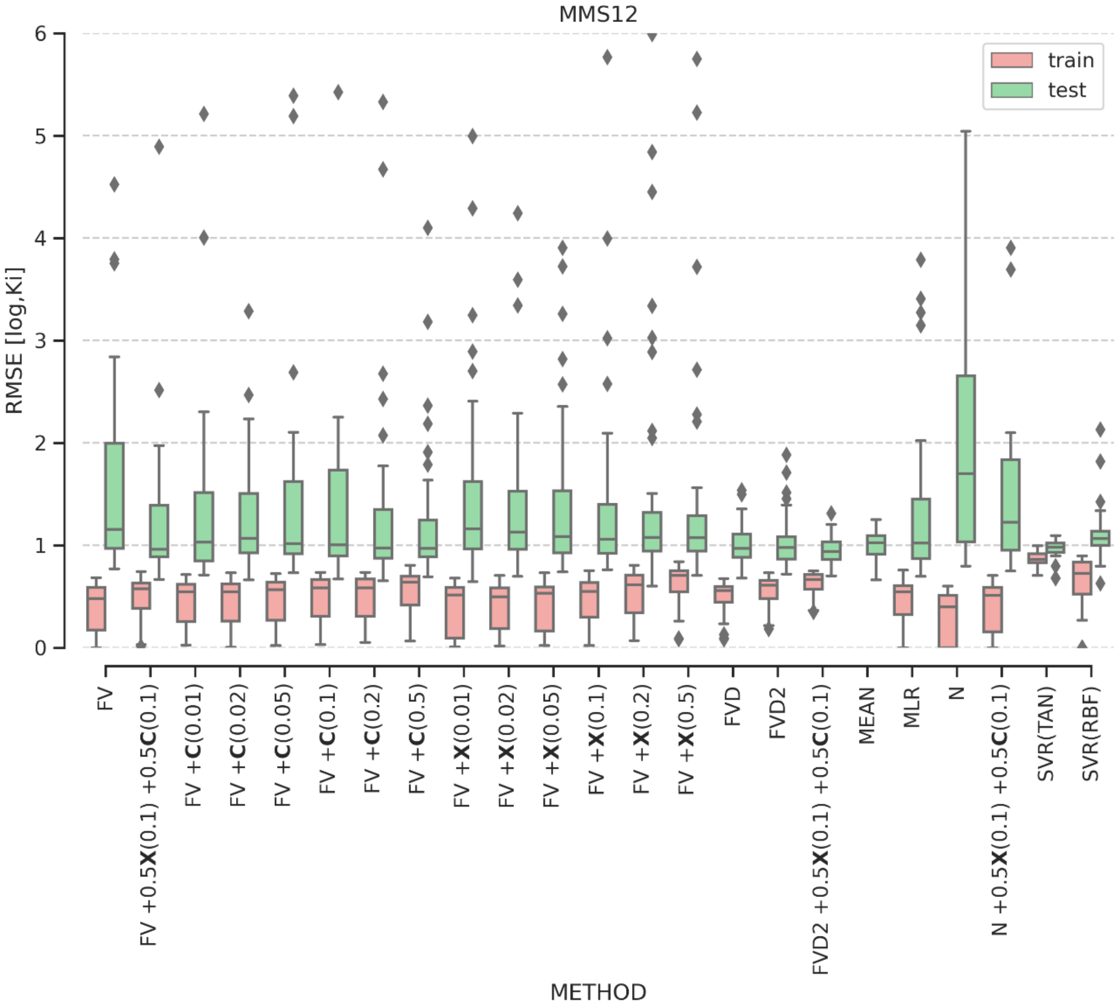

Figure S2. Comparison of prediction scores with various weights for STBL.
Boxplot comparisons of root mean squared error (RMSE) prediction scores for regression models derived using 23 methods, evaluated across 45 train-test data splits for 12 MMS datasets. The boxes represent the interquartile range, while the whiskers extend to the furthest datapoint within 1.5 times the interquartile range. Outliers are displayed as individual points. The method names with **X** or **C** represent that the methods are using the stability metric (**STBL**) for variables or coefficients, respectively. The number before each STBL letter (**X**, **C**) represents the magnitude of the STBL metric (λ), while the number in each bracket represents the magnitude of the perturbation (δ). The method name N represents SR method without any filters. TAN or RBF in the SVR methods is Tanimoto kernel[1,2] or RBF kernel, respectively. Nonlinear SVR with the RBF kernel has been extensively used for QSAR models. The SVR hyperparameters C, ε, and γ (only for

RBF kernel) were optimized by five-fold cross-validation of the training dataset.

References

1. RDKit: Open-source cheminformatics. https://www.rdkit.org doi:10.5281/zenodo.591637.
2. Pedregosa, F.; Varoquaux, G.; Gramfort, A.; Michel, V.; Thirion, B.; Grisel, O.; Blondel, M.; Prettenhofer, P.; Weiss, R.; Dubourg, V.; Vanderplas, J.; Passos, A.; Cournapeau, D.; Brucher, M.; Perrot, M.; Duchesnay, E. Scikit-learn: Machine learning in Python. J. Mach. Learn. Res. 2011, 12, 2825– 2830.
3. Takaki, K.; Miyao, T. Symbolic regression for the interpretation of quantitative structure-property relationships. Artificial Intelligence in the Life Sciences 2022, 2, 100046.
